# Actomyosin contractility as a mechanical checkpoint for cell state transitions

**DOI:** 10.1038/s41598-022-20089-8

**Published:** 2022-09-26

**Authors:** Saradha Venkatachalapathy, Dyuthi Sreekumar, Prasuna Ratna, G. V. Shivashankar

**Affiliations:** 1 Mechanobiology Institute, National University of Singapore, Singapore, 117411 Singapore; 2 Institute of Molecular Oncology, Italian Foundation for Cancer Research, 20139 Milan, Italy; 3 Department of Health Sciences and Technology, ETH Zurich, 8092 Zurich, Switzerland; 4 Division of Biology and Chemistry, Paul Scherrer Institut, 5232 Villigen, Switzerland

**Keywords:** Biophysics, Computational biology and bioinformatics, Image processing, Cell biology

## Abstract

Cell state transitions induced by mechano-chemical cues result in a heterogeneous population of cell states. While much of the work towards understanding the origins of such heterogeneity has focused on the gene regulatory mechanisms, the contribution of intrinsic mechanical properties of cells remains unknown. In this paper, using a well-defined single cell platform to induce cell-state transitions, we reveal the importance of actomyosin contractile forces in regulating the heterogeneous cell-fate decisions. Temporal analysis of laterally confined growth of fibroblasts revealed sequential changes in the colony morphology which was tightly coupled to the progressive erasure of lineage-specific transcription programs. Pseudo-trajectory constructed using unsupervised diffusion analysis of the colony morphology features revealed a bifurcation event in which some cells undergo successful cell state transitions towards partial reprogramming. Importantly, inhibiting actomyosin contractility before the bifurcation event leads to more efficient dedifferentiation. Taken together, this study highlights the presence of mechanical checkpoints that contribute to the heterogeneity in cell state transitions.

## Introduction

Cell state transitions are critical events in many biological processes including developmental programs, tissue maintenance, and disease progression^1–3^. A number of approaches have been developed to induce cell state transitions in cell culture models, including seminal experiments in which somatic cells were reprogrammed into stem cells in the presence of Yamanaka factors^4^. More recent experiments have shown that combining Yamanaka factors with the right mechano-chemical factors increased the efficiency of reprogramming^5–10^. Recent studies have also revealed the functional importance of cell mechanics in regulating the nuclear morphology, chromatin architecture and thereby gene regulatory networks. Such tight coupling between mechanical and biochemical properties of cells is exemplified in cell state transitions. In line with this, we demonstrated that the growth of cells on sustained geometric constraints can induce cell state transitions towards partial reprogramming^11^. These results highlight the tight coupling between cell mechanics and genome regulation that enables the induction of cell state transitions in vivo without exogenous factors, although the underlying mechanisms are poorly understood.

Several single cell RNA sequencing studies have revealed that cell state transitions are often asynchronous, leading to a large variability in cell-states at the population level^12–14^. In most transitions, there is an inherent heterogeneity reported i.e., the presence of distinct metastable cell states^15^. Current approaches that analyze such cell state transitions and the origins of the underlying heterogeneity highlight the contributions of transcriptional noise, clonal variability in stoichiometric ratios of the Yamanaka factors and variability in the physio-chemical microenvironment. However the role of cell mechanics and its coupling to chromatin organization in regulating the variability in cell state transitions has not been well explored. To this end, we use laterally confined growth induced cell state transitions as a model system to understand the role of cell mechanical states in driving such cell state transitions. Due to the well-defined initial boundary conditions of this platform, we would be able to study the cell state transitions arising from an initial population that is geometrically homogeneous. In addition, since the cells are confined, the cells in individual colonies at the end of the process would be from the same initial cell and would not have had any physical interactions with other colonies. Thereby, this platform controls for many technical sources of error and would be ideal for studying the inherent heterogeneity in cell state transitions.

In this paper, we map the cell state transitions that are induced by laterally confined growth. Sustained laterally confined growth of cells results in sequential changes to the nuclear morphology and chromatin organization with each cell division, leading to the progressive erasure of lineage-specific gene expression by inducing cell state transitions. We used colony morphology, combined with candidate gene expression markers for tracing the cell state transitions that occur during laterally confined growth. A pseudo-trajectory of laterally confined growth built using unsupervised diffusion analysis revealed a bifurcation event in which some cells undergo successful cell state transitions towards partial reprogramming. In addition we also observed that some colonies are proliferatively paused with time. These lead to variable growth kinetics patterns in the population leading to a morphologically heterogeneous population of colonies composed of cells in different cell states at day 10. Importantly we demonstrate, using pharmacological inhibitors, that the sub-cellular actomyosin contractility regulates cellular decisions during such cell-state transitions.

## Results

### Laterally confined growth of human fibroblasts induces cell-state transitions

**Figure 1:**
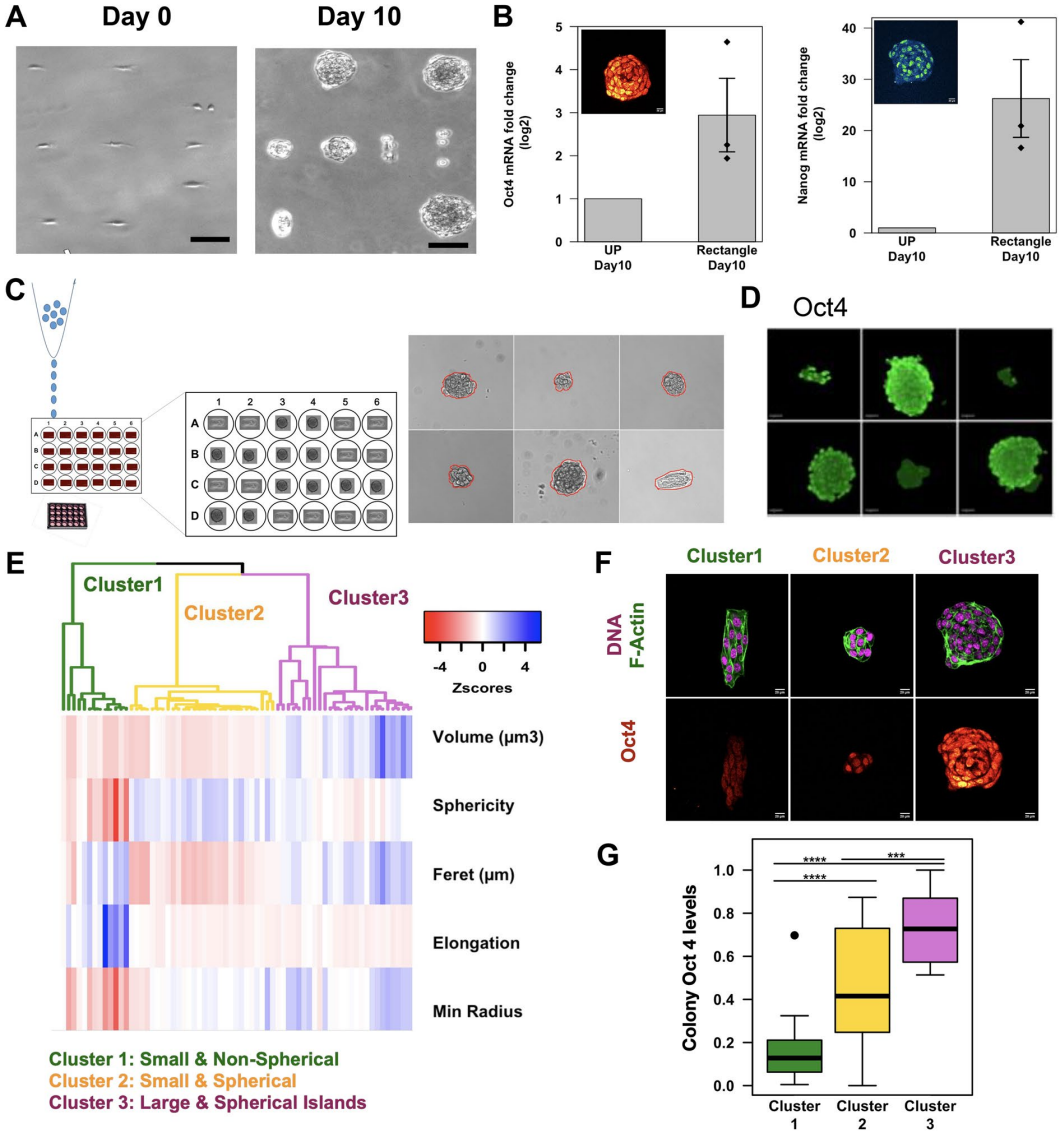
Laterally confined growth of fibroblasts induces cell state transitions: (**A**) representative images of HMF3A cells grown on rectangular patterns at day0, and day 10 of laterally confined growth. Scale bar is 100 microns. (**B**) mRNA levels of Oct4 (left) and Nanog (right) after 10 days of growth on rectangular pattern or unpatterned (UP) cells. Bar plots represent population means and standard error of means. n = 3 biological replicates (p < 0.05, one tailed Wilcoxon rank sum test—hypothesis was that the difference in the mRNA levels between the patterned and unpatterned condition was greater than 0). (Inset) Representative micrograph of a confocal slice of a colony at day 10 stained for Nanog (blue green) and Oct4 (heat colours). Scale bar is 20 μm. (**C**) Schematic describing the experimental setup. Single fibronectin island was stamped per well in a 24 well plate. Using FACs we seeded 1 to 2 cells per well. These were cultured for 10 days under normal culture conditions. (Right) Brightfield images showing colony formation within each well. Red lines are outlines of the colony. (**D**) Images of Oct4 staining within colonies (Green). Scale bar is 20 microns. These are representative results from 2 biological replicates. (**E**) Heatmap of selected morphological features of day 10 colonies, where each column in one colony. Dendrogram shows hierarchical clustering of these colonies resulting in 3 clusters. n = 68 colonies from 3 biological replicates. (**F**) Representative images from each cluster of day 10 colonies stained for DNA (magenta), F-Actin (green) and Oct4 (heat colours). (**G**) Boxplot of average Oct4 levels per colony across the 3 clusters. n = 68 colonies from 3 biological replicates. The Oct4 levels of each colony is treated as an independent measurement and the values are normalized within each biological replicate. Significance codes: ***p < 0.001 and ****p = 0.

Human mammary fibroblasts (HMF3A) were cultured on rectangular fibronectin micropatterns upto 10 days (see “Methods and materials” section). The surface was passivated such that cells continue to divide only on these rectangular islands, i.e. the growth was laterally confined. After 10 days of such laterally confined growth, single rectangular fibroblasts proliferate to form a colony of mostly spherical cells (Fig. 1A, Fig. S1A). This is evidenced by the increase in the area and circularity of the colonies over time (Fig. S1C,D, see “Methods and materials” section). To test whether the cells were indeed undergoing cell state transitions, we used the standard reprogramming markers: expression of Oct4 and Nanog and alkaline phosphatase activity^16,17^. Our control was unpatterned cells grown for 10 days i.e., without lateral confinement. We found that the transcript levels of Oct4 and Nanog were higher after 10 days of laterally confined growth (Fig. 1B). Further, we verified the protein level expression of these markers by performing immunofluorescence (Fig. 1B Insets). Remarkably, around 60% of the spheroids at day 10 were found to be positive for alkaline phosphatase activity whereas no cells were positive in the control (Fig. S1E–G). In line with previous studies^11,18^, these results indicate that laterally confined growth induces cell state transitions probably leading to partial reprogramming events in HMF3A cells.

We further wanted to check if laterally confined growth was induced only by the cell geometric constraints alone in the absence of any communication between colonies. To do so, we micropatterned a single fibronectin island in each well of a 24 well plate. We then used a cell sorter to seed 1 to 2 cells per well and wells which had exactly one rectangular cell after 3 h were cultured and monitored for 10 days (Fig. 1C). We found that single rectangular fibroblasts formed colonies of cells and expressed Oct4 (Fig. 1D). This indicates that laterally confined growth induced cell state transitions do not require any communication between neighboring colonies. These results further suggest that single cells under appropriate constraints can intrinsically be rewired to undergo cell-state transitions.

Interestingly, we observed a high degree of variability in both the alkaline phosphatase activity and sizes and shapes of single colonies (Fig. S1E), suggesting that the colony morphology is linked to its reprogramming state. In order to explore this further, we obtained high resolution 3D images of colonies at day 10 and observed variability in colony shape and size as well as their Oct4 expression levels (Fig. S1I,J). We quantified morphological features of colonies (see “Methods and materials” section), performed hierarchical clustering and obtained 3 clusters of colonies (Fig. 1E). Colonies belonging to Cluster 3 are large and spherical whereas those belonging to Cluster 2 are small and spherical and finally Cluster 1 are small elongated colonies (Fig. 1F). Amongst these clusters, we find that colonies belonging to Cluster 1 and Cluster 3 have lowest and highest levels of Oct4 respectively (Fig. 1G). Consistently, we found that large circular colonies have the highest alkaline phosphatase activity (Fig. S1H). These results suggest that the morphology of the colonies is coupled to its reprogramming efficiency.

### Time course kinetics reveal a tight coupling between colony morphology and protein expression levels

**Figure 2:**
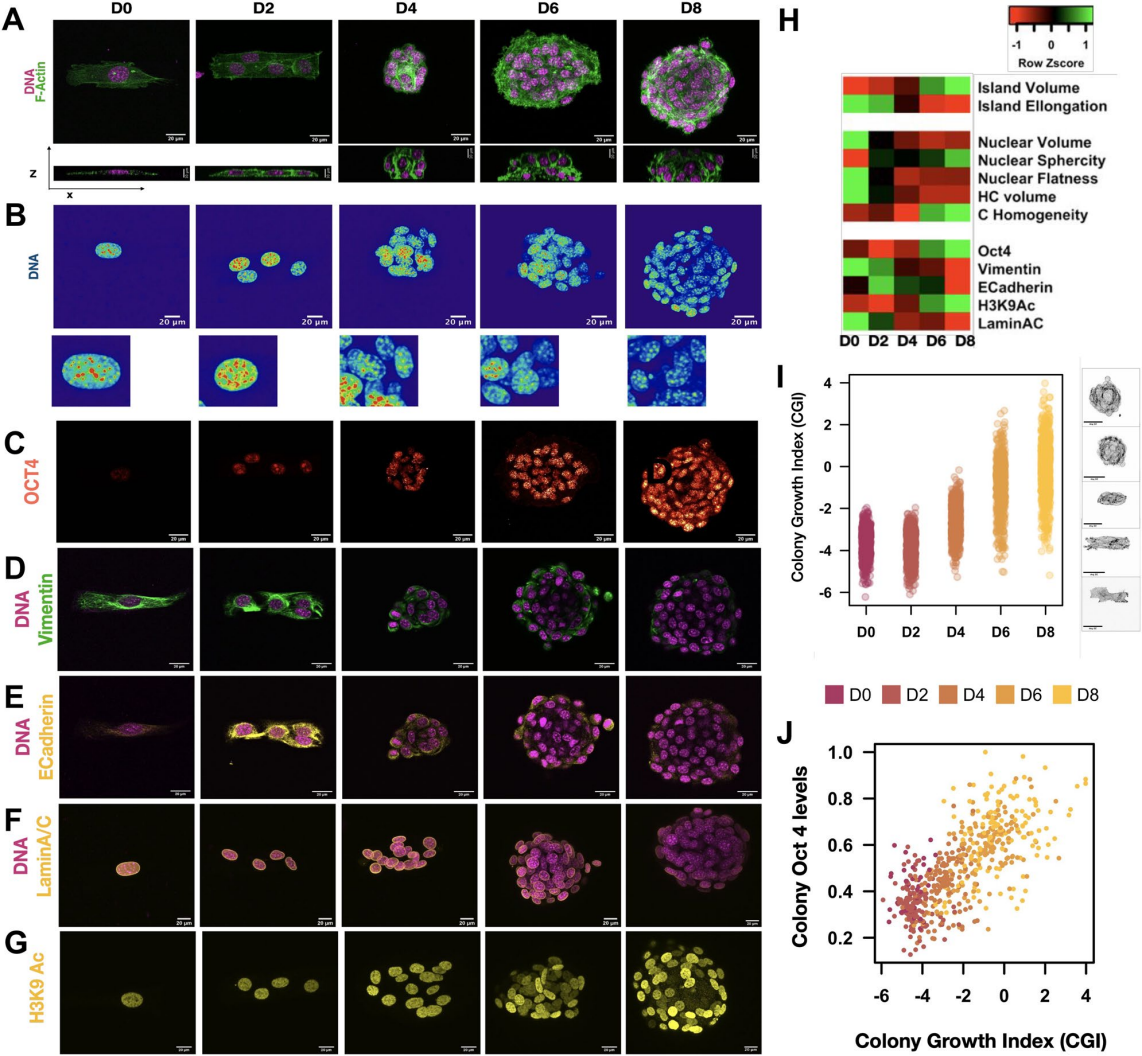
Time course kinetics of reprogramming events demonstrates the coupling between physical and biochemical features: (**A**) representative images colonies stained for DNA (magenta), F-Actin (green) from day 0 to day 8. Bottom row contains an XZ orthogonal slice (Scale bar = 20 μm). (**B**) Representative images of colonies stained for DNA from day 0 to day 8 (Scale bar = 20 μm). Bottom row contains a 17 μm × 17 μm zoomed in section. Representative images of colonies stained for Oct4 (heat colors) (**C**), DNA (Magenta) and Vimentin (green) (**D**), E-Cadherin (yellow) (**E**) Lamin AC (**F**) and H3K9Ac (Yellow) (**G**) from day 0 to day 8. (Scale bar = 20 μm). (**H**) Heatmap showing the changes in the population means of reprogramming markers island morphometrics and chromatin features at various time points. n ≥ 3 replicates for each measure. The values are normalized within each biological replicate. (**I**) Jitter plot of colony Growth Index (CGI) from day 0 to day 8. Each dot represents one colony. Right: Collage of z projected colonies stained for F-Actin arranged in increasing order of CGI. n = 3493 colonies from 5 biological replicates. (**J**) Scatter plot of Average Oct4 levels against Island Growth Index per island from Day 0 to Day 8. Each dot represents one island. n = 1541 colonies from 3 biological replicates. The values are normalized within each biological replicate.

We next asked if the morphological features of colonies could be used to map the biochemical features (e.g. protein expression) during laterally confined growth. To this end, we imaged individual colonies stained for key proteins from day 0, i.e., 3 h after initial seeding, to day 8 at high resolution (Fig. 2A–G). This enabled us to compute protein expression levels, morphological features of colonies as well as architectural features of individual nuclei from each colony. Figure 2H summarizes the sequential changes that occur to the structural and proteomic landscape during the reprogramming process at the population level.

In order to quantitatively describe the structural changes of the colonies undergoing laterally confined growth, we measured multiple morphological features from 3D confocal images (Fig. 2A). Notably, we observed an increase in colony volume and a decrease in colony elongation indicating the formation of 3D spheroids with time. In order to succinctly describe these temporally relevant changes, the multivariate colony morphology feature space was reduced using Linear Discriminant Analysis resulting in a single feature vector namely Colony Growth Index (CGI). The CGI is small for small, elongated colonies (Fig. S2A) and during laterally confined growth CGI’s value and variability increases (Fig. 2I). Hence although the initial population is morphologically homogeneous, the variability sequentially increases resulting in a heterogeneous population by day 10.

We next assessed the changes in the cell’s structure that accompany the formation of spheroids during laterally confined growth. To do so, we segmented individual nuclei within each colony and we have visualized the changes in cell shape as represented by the voronoi spaces within the colonies during laterally confined growth (Fig. S2B). In our platform, single fibroblasts in each rectangular colony divide to form a spheroid of multiple cells by day 8. Repeated cell divisions on a small matrix area results in cells having lower cell–matrix contact and more cell–cell contacts and becoming more isotropic (Fig. S2C,D). To further support this, we see that the cells exhibit more beta-catenin expression between the cells after 4 days of culture (Fig. S2F). Interestingly, in these spheroids, the cells in the bottom are attached to the matrix and display focal adhesions and in the rest of the structure, cells form cell–cell junctions (Fig. S2G). Further, we observe a progressive change in the actin structure—from prominent parallel actin stress fibers that run along the cell length at day 0 to a more cortical-actin along the cell–cell interfaces at day 10 (Fig. S2F). Hence with each cell division, the cells lose their matrix attachment, form more cell–cell junctions and reorganize their actin stress fiber organization which cumulatively results in the formation of spheroids.

Since nuclear morphology and chromatin organization have been shown to be important markers of cell state transitions^19^, we next measured multiple features that exhaustively describe them (Fig. 2B). During laterally confined growth, we observed that the nuclei become smaller, more spherical and less flat. In addition, the chromatin became less condensed, as supported by the decrease in Heterochromatin (HC) volume and higher Chromatin (C) Homogeneity at later time points (Fig. 2H). The multiple single nuclear features were combined to obtain a composite feature namely Chromatin Reorganization Index (CRI) which increases in value and variability during laterally confined growth (Fig. S2H). Small nuclei with a homogeneous DNA texture have high CRI values (Fig. S2I). Further, the chromatin reorganization index (CRI), which is a combination of multiple nuclear morphology and chromatin organization features, is predictive of the cellular Oct4 levels (Fig. S2J). Therefore, one can potentially use CRI to describe the changes in the chromatin organization that occur during laterally confined growth.

In conjunction with these morphological changes, we observed marked differences in the expression levels of key markers of cell state transitions (Fig. 2C–G, Fig. S2K). In line with our previous results, there was a progressive increase in the expression of the reprogramming marker Oct4. Along with this we also observed a decrease in the expression level of the mesenchymal protein, vimentin, and a transient increase in the levels of epithelial marker E-cadherin, indicating that some cells are undergoing mesenchymal to epithelial transitions^1^. In addition, the nucleus became softer, and the chromatin more relaxed as seen by the decrease in nuclear LaminA/C levels and an increase in H3K9Ac levels with time^20,21^. Taken together, these results reveal that laterally confined growth induces cell state transitions often associated with partial reprogramming.

In addition, to show a clear relationship between the chromatin structure, obtained using imaging, and the epigenetic landscape, we computed the correlation between characteristic nuclear morphology and chromatin organization features and the cellular levels of H3K9Ac (Fig. S2L). As expected, we observe that the heterochromatin (HC) volume is negatively correlated with H3K9Ac levels. Further, cells with higher H2K9Ac levels have less random chromatin textures that are more uniform over large length scales as indicated by the texture features entropy and correlation respectively. These results are inline with the literature that state that the chromatin organization is tightly linked to its epigenetic state^6^.

Next, we aimed to check if there was a relationship between the colony structure and protein expression space. To this end, we used linear regression to model the relationship between the mean protein levels per colony and the CGI across time points. For instance, the mean Oct4 level per colony varies linearly with the CGI across all time points and hence, the model was able to predict the Oct4 expression (Fig. 2J, Fig. S2M). This has been done for all the aforementioned marker proteins as well as the mean CRI of the nuclei in a given colony. To assess our model, we measured the coefficient of determination (R.squared) and the Pearson correlation coefficient (R) between the actual and predicted values for a test dataset, which was unseen by the model. Overall, correlation coefficients between the actual and predicted protein levels were high for most proteins (Fig. S2N). Hence, the morphological properties of the colonies could sufficiently describe the changes in expression profiles of key proteins that describe cell states. While CGI could explain the general direction of changes in protein expression profile of cells in the colony, the model only accounted for 40–60% of the observed variance (as estimated by R.squared). This indicates that there might be other factors that also determine protein expression.

Collectively, we observed that laterally confined growth induces changes in the chromatin structure as well as the protein expression profile. Importantly, although we started with a morphologically homogeneous population of single cells, the variability sequentially increases culminating with the heterogeneity in colony structure and protein expression levels seen at day 10. Since, the structure of the colony is correlated to its protein levels, we next aimed to model the trajectory of cell state transitions during laterally confined growth using the morphological properties of colonies.

### Diffusion analysis reveals stages of laterally confined growth

**Figure 3:**
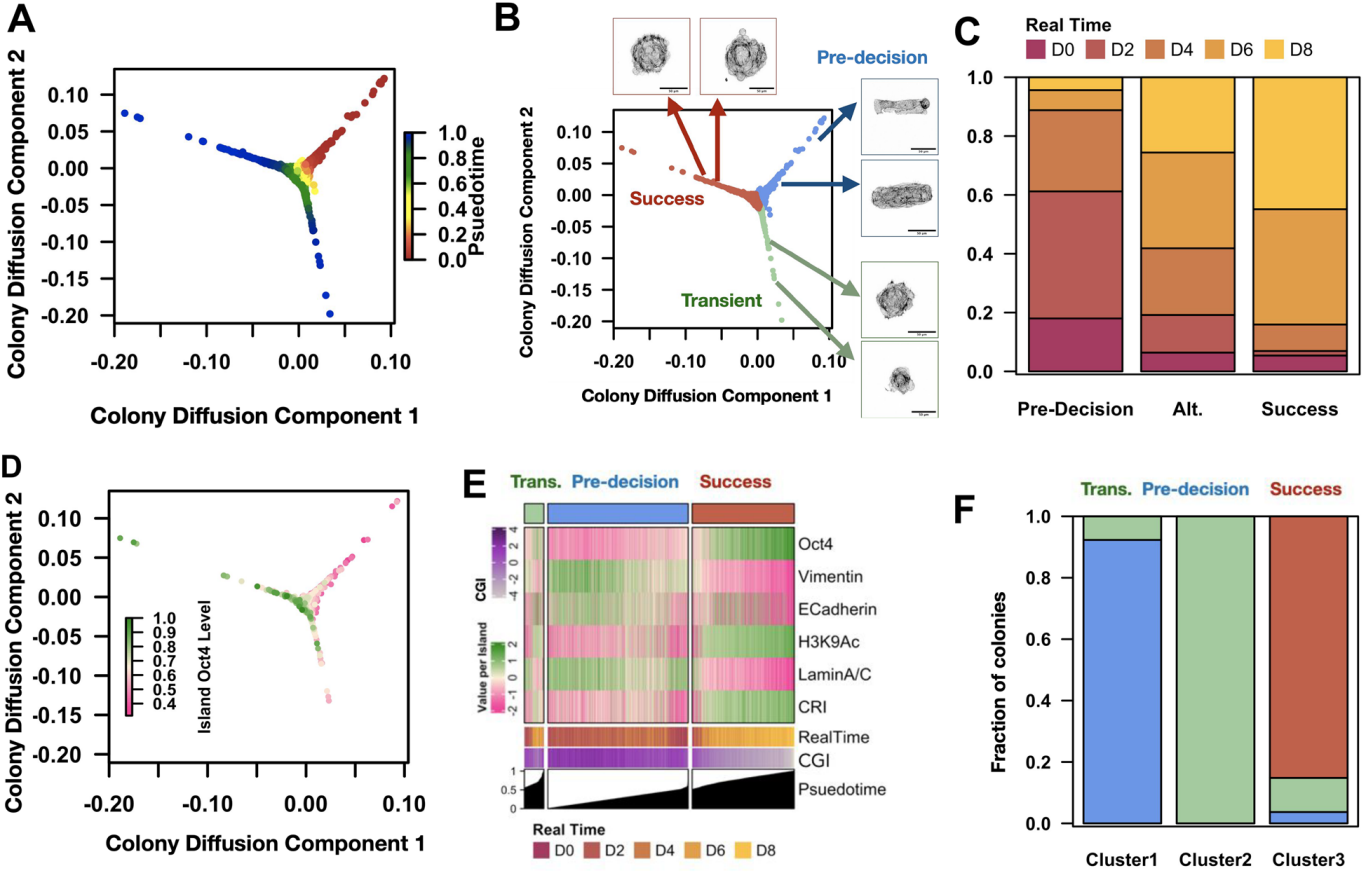
Diffusion analysis reveals stages of laterally confined growth: (**A**) Diffusion Map of colonies from Day 0 to 8 color coded with the inferred pseudotime. The sequential color indicates the position of each cell in pseudotime starting from 0 to 1. ~ 3000 colonies were used in total from 6 biological replicates. (**B**) The branches are defined as Pre-decision (blue), Transient (Trans.) (green) and Success (maroon) separately. Representative micrographs of images of the various branches are displayed along the margins. The arrows indicate their approximate location. Scale bar = 20 µm. (**C**) The branch membership fraction of colonies from Day 0 to Day 8. The color code is displayed along the top margin. (**D**) Diffusion Map of colonies color coded with the average Oct4 levels per colony. n = 3 biological replicates. The values are normalized within each biological replicate. (**E**) Heatmap depicting the branch-wise protein expression and Chromatin Reorganization Index (CRI) of colonies ordered with increasing pseudotime. Note that each column is a colony. The first column-side color bar represents the real time of the colonies between Day 0 and 8. The second column-color bar represents the Colony Growth Index (CGI) values of the colonies. The histogram at the bottom panel depicts the corresponding pseudotime value of the colonies (see “Methods and materials” section). (**F**) The branch membership fraction colonies of the three D10 clusters identified Fig. 1E. n = 68 colonies from 3 biological replicates.

In recent years, multiple methods have been developed to order cells along a pseudo-trajectory using high dimensional snapshot time series data. These methods assume cells that are closely related (in a lineage) will have similar features^14,22,23^. Here we leveraged such approaches to trace laterally confined growth using image-derived features. To this end, we built an unsupervised diffusion map using just the morphological features of colonies to obtain a pseudo-trajectory of laterally confined growth (Fig. 3A, see “Methods and materials” section). The position of colonies in real time is shown in Fig. S3A,B. Consistent with our previous result, the colonies at day 0 are homogeneous as seen by their clustered positioning in the diffusion map. In contrast, colonies between days 4 and 8 exhibit considerable variability. Further, we identified three branches in the obtained pseudo-trajectory (Fig. 3B). We named them pre-decision, success and transient based on their properties. The pre-decision branch was populated with small, elongated colonies mainly from days 0 to 4. The success branch consisted of large spherical colonies, mainly composed of colonies from days 6 and 8. Finally the transient branch consisted of small spherical colonies between days 4 through 8 (Fig. 3C, Fig. S3C). We next asked how the protein expression profile changes along these branches. The colonies that took the successful path had higher Oct4 levels compared to those that took the alternate path (Fig. 3D). In contrast to the transient branch, the colonies that belong to the success branch had lower Vimentin and E-cadherin levels indicating a reduction in both epithelial and mesenchymal cell states. We also find that the colonies in the success branch have lower LaminA/C levels and higher H3K9Ac levels suggesting that the nuclei are much softer, and the chromatin is less condensed as supported by the higher CRI levels in these colonies (Fig. 3E).

Further, we measured the variability in the reprogramming efficiency within each colony after the bifurcation event around Day 4. We quantified this variability by measuring the coefficient of variation of Oct4 levels of cells within a colony for colonies belonging to the success and transient branch. We observe that colonies belonging to the success branch have a more homogenous population across both timepoints (Fig. S3D). However, we would like to point out that the most striking difference between the transient and success branch is the size or number of cells per colony: the colonies belonging to the success branch are populated by more number of cells and hence are larger (Fig. S3E). Based on these observations, we hypothesize that the cells in the “transient” colonies have stopped proliferating, indicating that they might be stuck in a local energy minima in the Waddington energy landscape. Hence, the cells from the initial population of fibroblasts (pre-decision) either take a successful path towards partial reprogramming or get paused at a transient stage.

To evaluate the robustness of the trajectory inferred from snapshot data from days 0 to 8, we next asked if the three clusters obtained at day 10 (described in Fig. 1E) belonged to different branches. To that end we obtained the diffusion coefficients and the branch membership of the colonies at day 10. We found that each of three clusters had almost exclusive membership in one of the 3 branches. Pre-decision branch was almost entirely made up of colonies from Cluster 1, the success branch was mainly composed of those from Cluster 3 and the transient branch was exclusive to Cluster 2 colonies (Fig. 3F). Therefore, we conclude that the bifurcation in the pseudo-trajectory of laterally confined growth can be used to explain the origins of Clusters 2 and 3 at day 10. Collectively, these results revealed the trajectory of laterally confined growth that correlated with cell-state changes.

### Acto-myosin contractility regulates cellular decisions during laterally confined growth

**Figure 4:**
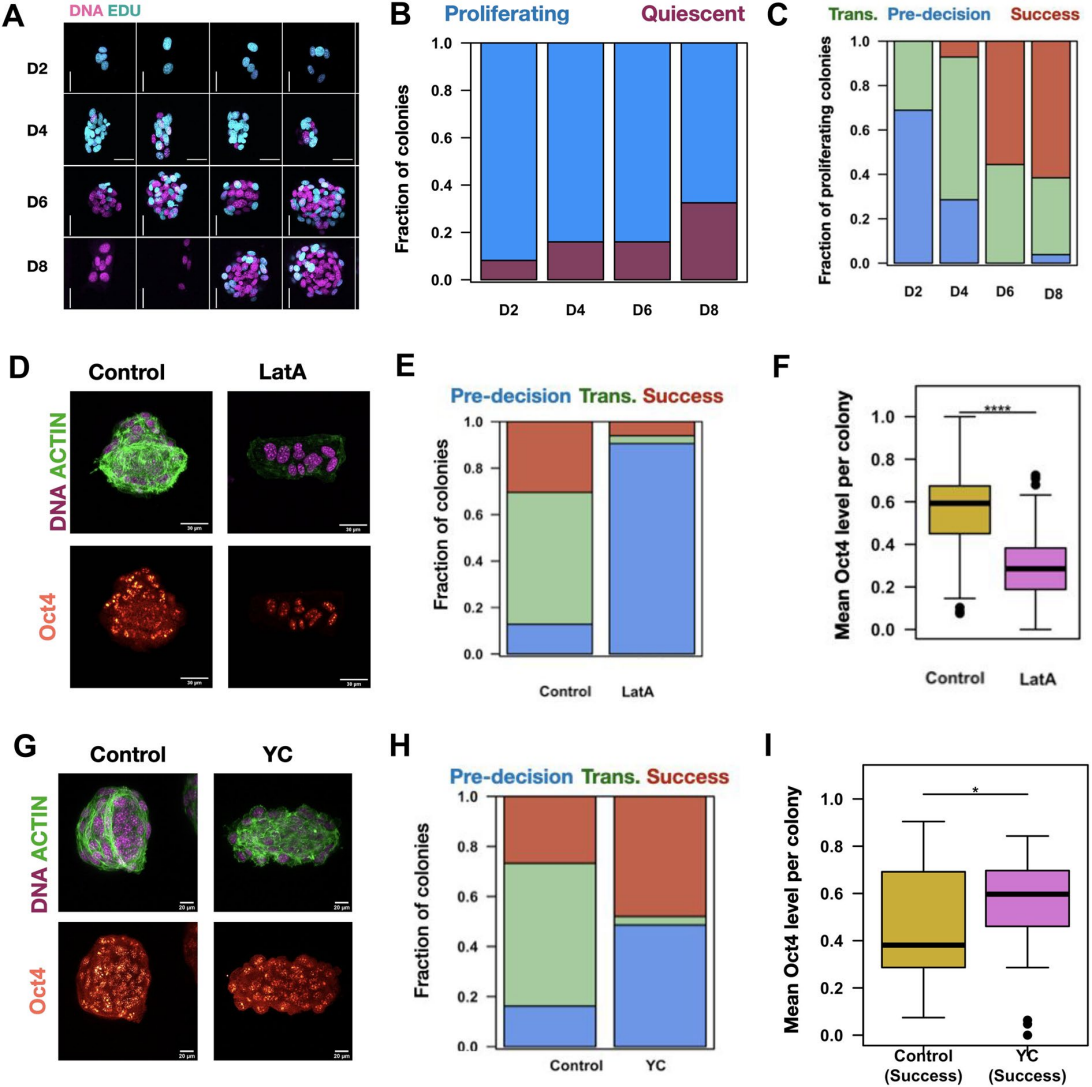
Acto-myosin contractility regulates the cellular decisions during laterally confined growth: (**A**) montages of Edu labeled colonies from day 2 to 8. Edu (Cyan) and DNA (Magenta). Scale bar = 20 microns. (**B**) Stacked barplot of the fraction of colonies that are Proliferating. Colonies with at least one Edu positive nucleus are identified to be Proliferating (blue) and the rest are labeled Quiescent (orchid). (**C**) The branch membership fraction of Proliferating colonies from day 0 to day 8. The color code is displayed along the top margin. (**D**) Representative images of control and Latrunculin A (LatA) treated colonies at day 10. F-Actin (Green), DNA (Magenta) and Oct4 (Heat Colors). Scale bar = 20 μm. (**E**) The branch membership fraction of colonies under the two culture conditions. The color code is displayed along the top margin. (**F**) Mean Oct4 Levels of control and Latrunculin A (LatA) treated colonies at day 10. n = 3 biological replicates, p < 0.0001. (**G**) Representative images of control and Y Compound (YC) treated colonies at day 10. F-Actin (Green), DNA (Magenta) and Oct4 (Heat Colors). Scale bar = 20 μm. (**H**) The branch membership fraction of colonies under the two culture conditions. The color code is displayed along the top margin. (**I**) Mean Oct4 Levels of control and Y-Compound (YC) treated colonies that belong to the success branch at day 10. p < 0.05 n = 3 biological replicates.

The continued existence of colonies in the pre-decision branch at days 6, 8, and 10 (cluster1) suggests that there are colonies whose proliferation is paused. In order to test this, we used EdU (5 ethynyl 2ʹ deoxyuridine), which is a thymidine analogue, to label cells that had proliferated during the last 24 h (Fig. 4A). We observed an increase in the fraction of non-proliferating colonies, i.e., colonies with no Edu positive nuclei, from 8% of colonies at day 2 to 32% of the colonies at day 8 (Fig. 4B). Consistently, the mean colony levels of Ki67, a known cellular marker for cell proliferation also decreases with time (Fig. S4A,B). Hence, in the beginning most of the colonies were proliferating but after day 4, some colonies stopped. This coincides with the bifurcation event observed in the pseudo-trajectory around day 4 (Fig. 3). We found that the colonies that continue to proliferate have higher CGI at day 8 but this was not the case at earlier time points like days 2 and 4 (Fig. S4C). More importantly, we found that the majority of the colonies that are proliferating at day 8, belong to the success branch (Fig. 4C). Therefore, colonies that continue to proliferate after 6 days of laterally confined growth likely belong to the success branch. To check if this was true, we plotted the proliferation (Edu) status of the colonies after the branching point at day 4. In line with our hypothesis, we find that the colonies in the success branch continue to proliferate whereas those in the transient branch do not (Fig. S4D,E). Hence, we posit that the variable growth kinetics induced by proliferative pausing contributes to the heterogeneity observed in the colony structure over time.

Since we observed a strong correlation between colony structure and cell state transitions, we next asked if we could tune the efficiency of reprogramming (as indicated by the levels of Oct4) by targeting the intrinsic cellular properties that control colony structure. Filamentous Actin (F-Actin) and phosphorylated Myosin Light Chain (pMLC) define actomyosin contractility and consequently are a major driver of structural properties of cells^24,25^. Furthermore, we found that while the F-Actin levels in colonies increased in both the success and transient branches, the pMLC levels were relatively higher in the success branch (Fig. S4F,G). These findings suggest that actomyosin contractile forces could play a differential role in cellular decisions around days 2 to 4. Therefore, we asked if tuning cell mechanics by targeting actomyosin contractility would alter the growth patterns and reprogramming levels. Since much of the heterogeneity sets in between days 2 and 4, we decided to intervene from day 2.

We tested the relevance of F-Actin in laterally confined growth using Latrunculin A to keep actin in a depolymerized state between days 2 through 10 (Fig. 4D, Fig. S4H). As expected, the treatment led to a decrease in F-Actin and pMLC levels (Fig. S4I,J). Additionally, in the treated sample we observed smaller and more elongated colonies suggesting that treatment interfered with the proliferation and formation of spheroids (Fig. S4K,L). This is further supported by the observation that colonies from the treated sample have lower CGI (Fig. S4M) and most of the colonies in the population remain in the pre-decision branch (Fig. 4E). Importantly, we saw a sharp decrease in the expression of Oct4 (Fig. 4F). These results demonstrate that F-actin is important for cell proliferation and formation of large spherical colonies and thereby, the reprogramming process.

To differentiate the contribution of F-Actin to that of pMLC, we treated the cells with Y27632 (Y-Compound), which is an inhibitor of Rho associated protein kinase II (ROCKII). Although we observed a decrease in the F-Actin and pMLC levels after Y-compound treatment, actin filaments persisted (Fig. 4G, Fig. S4N–P). Additionally, the treatment led to an increase in colony volume (Fig. S4Q), which is in line with studies that have shown that ROCK inhibition can lead to higher cell proliferation^26^. However, these larger colonies were elongated (Fig. S4R). This highlights the role of actomyosin contractility in maintaining the shape of the colony. Importantly, after Y compound treatment, the fraction of cells in the transient branch reduced drastically, while there was an increase in the number of colonies in the success branch (Fig. 4H). At the population level, we did not observe a significant difference in the Oct4 levels between control and treated samples (Fig. S4T). To remove the variability introduced by colonies in the pre-decision branch, we compared control and Y-compound treated colonies belonging to the success branch and observed that ROCK inhibition leads to an increase in Oct4 levels by almost 20% (Fig. 4I). Hence, reducing actomyosin contractility promotes the formation of large colonies and successful reprogramming. Collectively, these results indicate that either tuning actomyosin contractility can alter the proliferative capacity and efficiency of cell state transitions during laterally confined growth. To further dissect the role of cell proliferation during the reprogramming process, we next aimed to check the effects of directly targeting cell proliferation by exposing the cells to nocodazole, an antimitotic agent that targets microtubule polymerization, from 2 to 10 days of culture. As expected, we observed that following this perturbation, the colonies remain small and elongated and the cells also do not reprogram (Fig. S4U,V). Taken together, these results indicate that the cell mechanics can affect the reprogramming efficiency of laterally confined growth and one way this is achieved is via controlling cell proliferation.

## Discussion

Cell state transitions are often accompanied by changes to the gene regulatory networks, transcription profile, 3D chromatin organization, and cell morphology. These transitions have classically been described using Waddington’s epigenetic landscape^27^ and a number of theoretical models have described the transient metastable states that are associated with these transitions^28,29^. For instance, during reprogramming, differentiated cells have to overcome multiple barriers such as Mesenchymal to Epithelial Transitions (MET). These events are brought about by active forces that alter the cell morphology and chromatin architecture and thereby gene expression programs. Many studies have identified microenvironmental cues^8,30^ as well as subpopulations of somatic cells^31^ that promote more efficient cell state transitions. Importantly, laterally confined growth of single fibroblasts under certain geometric constraints has been shown to induce more efficient cell state transitions^11^. In addition, single cell experiments have revealed that cell state transitions lead to heterogeneous outputs and this is one major reason for low efficiencies of cellular dedifferentiation. In this study, using a well defined experimental model, which allows us to track cell state transitions arising from one cell over time, we reveal a role for actomyosin contractility in regulating cell-fate decisions that contribute to such variability.

Here we first demonstrate that the laterally confined growth of human fibroblasts on geometric constraints induces cell state transitions. Temporal evaluation of the protein expression levels in each colony revealed that these colonies express classical markers of reprogramming. These include a reduction in the expression of somatic cell markers, a transient MET, and progressive reorganization of the chromatin structure, all ultimately culminating in the expression of early reprogramming markers. Concomitantly, we also observed changes in the colony morphology from an elongated single cell to a colony of mostly spherical cells. In our system, we start with a stretched elongated cell on a stiff fibronectin matrix that divides on top of one another to form a colony of spherical cells. The colonies, hence progressively acquire cells that have less actin stress fibers, softer nuclei with more open chromatin that are more circular in shape. Overall these changes indicate that the colonies are going from stiffer cell state to a softer cell state. Interestingly, we observed that the formation of cell–cell contacts are accompanied by the frustration of actin stress fibers and enrichment of actin at the cell–cell junctions during laterally confined growth of fibroblasts. This observation suggests that the reorganization of the actin structure plays an important role in the formation of spheroids as well as cell state transitions induced by latterly confined growth. In line with this hypothesis, we find that by targeting actin polymerization and/or actomyosin contractility, one could tune both the structure of the colony as well as the cell state transitions. Importantly, we found that the colony morphology and the protein expression of its cells were coupled. This enabled us to use colony morphology as a succinct and convenient reflector of the cell state transitions that occur during laterally confined growth.

**Figure 5:**
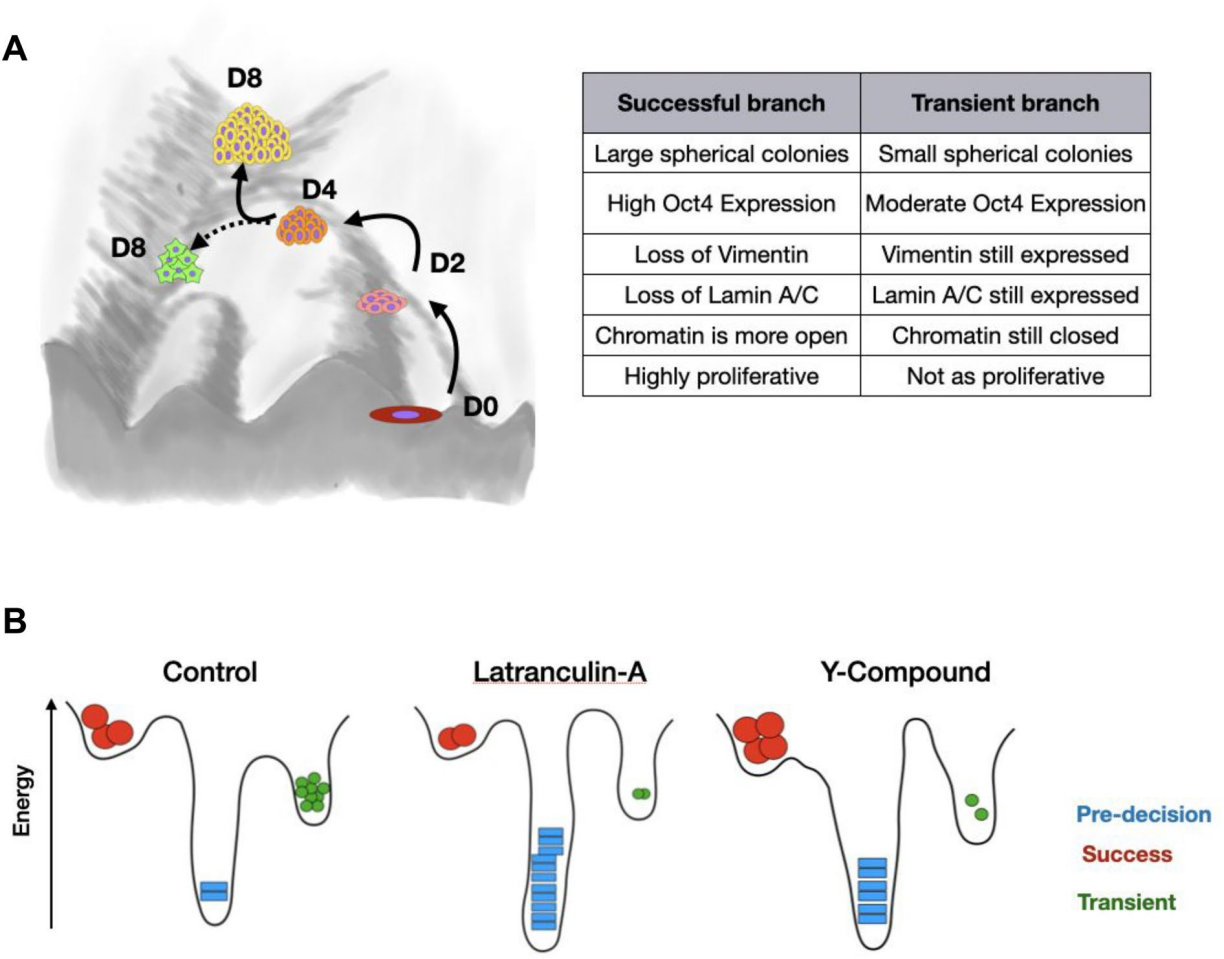
Graphical summary. (**A**) Graphical illustration of laterally confined growth induced cell state transitions: during laterally confined growth, a colony of cells metaphorically traverses through Waddington’s energy landscape. Two subpopulations were identified: a successful one with efficient cell state transitions and a transient branch with a mixture of cell states that are likely stuck in a local energy minima. The table on the right summarizes the key points of differences between the two trajectories. (**B**) The energy required to make certain cell state transitions can be modulated by the intrinsic cellular property of the actin cytoskeleton. Thereby enabling the direction of cellular decisions that occur during cell state transitions.

Even though the initial population was geometrically homogeneous, after 10 days of laterally confined growth we observed a morphologically heterogeneous population of colonies composed of cells in different cell states. To understand the temporal evolution of such variability, we first computed a pseudo-trajectory of laterally confined growth using the colony morphology features. Such methods have been extensively used in single cell RNA seq experiments to infer the trajectories of important processes such as differentiation and reprogramming^14,22,23^. Such an approach revealed a bifurcated trajectory in which, around day 4 colonies either took the successful path resulting in large spheroids composed of cells with high reprogramming levels, or an alternate path in which they ended up as small, circular colonies (Fig. 5A). In addition, we observed progressive proliferative pausing with time, starting around day 4. Only the cells undergoing successful cell state transitions continued to proliferate, indicating that this is a sequential process where the proliferation and reprogramming potential are coupled. These results indicate the presence of certain check points and that cellular decisions regarding cell state transitions occur between days 2 and 4 of laterally confined growth. Therefore, to control the cell state transitions in our platform to obtain better reprogramming efficiency, we perturbed the system right before the variability in the population sets in (day 2).

In our platform cells are growing in 3D and with each cell division, the nucleus is becoming softer and the chromatin more open. Such properties are characteristic of stem cells, which are soft and transcriptionally disengage rigidity sensing mechanisms^21,32^. Therefore, we targeted the sub-cellular properties that control rigidity: Filamentous Actin (F-Actin) and phosphorylated Myosin Light Chain (pMLC). We perturbed the cells after 2 days of culture before the bifurcation event. Inhibiting the F-Actin polymerization via Latrunculin treatment led to an arrest in cell division and even though cells survived, they did not undergo cell state transitions and were stuck in the pre-decision stage. In contrast, reducing actomyosin contractility via Y-Compound treatment led to increased proliferation and successful cell-state transitions in a subset of colonies, while reducing the number of colonies that are in the transient stage. These results indicate that certain cell state transitions can be modulated by tuning the actomyosin contractility and thereby enabling the direction of cellular decisions that occur during cell state transitions (Fig. 5B).

Taken together, our results highlight the usefulness of image derived features in capturing cell state specific information and also underscores the role of cell mechanics in modulating cellular decisions during cell state transitions. They further indicate the presence of mechanical checkpoints in cell state transitions and demonstrate how we can bypass them via interventions. Identifying the molecular pathways involved in these checkpoints require further exploration. One possible candidate is the Hippo/YAP pathway which is both mechanosensitive as well as an important modulator of cell proliferation. These studies open interesting possibilities of applying targeted mechanical interventions to improve the efficiency of cell state transitions.

## Materials and methods

### Microcontact printing

Fibronectin micropatterning was carried out. Polydimethylsiloxane (PDMS) elastomer (SYLGARD 184; Dow Corning) was used in a 1:10 ratio of curative-to-precursor according to the manufacturer’s protocol. This mixture was poured into microfabricated silicon wafers. These wafers were degassed for 45 min. To prepare stamps, the wafers were kept in the oven at 80 °C for 3 h. Freshly made PDMS stamps were oxidized and sterilized under high power in Plasma Cleaner (Model PDC-002; Harrick Scientific) for 5 min. 10% fibronectin solution was allowed to adsorb onto the surface of each PDMS stamp under sterile condition. The PDMS stamp was then deposited onto the surface of hydrophobic dishes (uncoated dish from ibidi) to allow transferring of the micro features. The surface was then treated with 2 mg/mL Pluronic F-127 (Sigma) for 30 min to passivate non–fibronectin-coated regions. Rectangles with an area of ~ 3300 μm^2^ (width 29 µm and length 116 µm) were stamped on uncoated Ibidi dishes. These micropatterned dishes were then passivated with 0.2% pluronic acid (Sigma P2443) for 5 min and washed twice with 1× PBS before cell seeding.

### Cell culture and drug treatment

Human fibroblast cells HMF3A cells (ATCC) were grown in DMEM (Gibco; Life Technologies), supplemented with 10% (vol/vol) FBS (Gibco; Thermo Fisher Scientific) and 1% penicillin–streptomycin (Gibco; Thermo Fisher Scientific). Cells were maintained in 5% CO2 at 37 °C. Cells were trypsinized and seeded on fibronectin-micropatterned dishes at a concentration of ~ 7000 cells/mL. Cells on the micropatterns were grown in previously described culture media for up to 10 days at 37 °C and 5% CO2. The media was replaced with fresh culture media on every alternate day. Within 10 days of culture on micropatterns, the majority of cells formed tightly packed multicellular spherical bodies (spheroids). Actin filaments were depolymerized with 200 nM Latrunculin A (Sigma). Rho-associated protein kinase II (ROCK) activity was inhibited by treating cells with 20 μM Y-27632 (Sigma). Nocodazole (Sigma) was used at a concentration of 5 µg/mL. Drugs were added from day 2 and the media with or without the drugs was replenished every alternate day.

### Immunofluorescence assay

Cells were fixed in 4% Paraformaldehyde (Sigma) in PBS buffer (pH 7.4) for 15 min, followed by washing with PBS (5 min × 3). Cells were permeabilized using 0.5% Triton (Sigma-Aldrich) in PBS for 15 min. After incubating with blocking solution [5% BSA (A3059; Sigma-Aldrich) in PBS] overnight at 4 °C, cells were incubated overnight at 4 °C with the primary antibodies diluted in the blocking solution: Oct4 (1:200; ab18976), Nanog (1:150;CST, #4903), Phospho-Myosin Light Chain 2 (1:200; CST, #3671), Ki67 (1:200; CST, #9129),E-Cadherin (1:200; ab76057),Vimentin (1:200; CST, #5741),α-Tubulin (1:200; ab15246),LaminA/C (1:200; CST, #4777),H3K9Ac (1:200; ab441). Cells were washed with PBS (10 min × 3) and incubated with corresponding fluorescent-labeled secondary antibodies diluted in blocking solution for 2 h at room temperature, followed by washing with PBS (10 min × 3). The nucleus was stained with NucBlue Live ReadyProbes (Molecular Probes; Thermo Fisher Scientific) in PBS for 20 min at room temperature, and filamentous actin was stained using phalloidin Alexa Fluor 488 or 568 or 647 (1:100; Molecular Probes; Thermo Fisher Scientific) for 1 h.

### Alkaline phosphatase activity assay

Cells and spheroids were fixed with 4% Paraformaldehyde (Sigma) at room temperature for 30 min. This was followed by washing with 1X PBS and Tris buffered saline (100 mM Tris and 5 mM MgCl2 in deionized water, pH 7.4). Further, the cells and colonies were incubated with the alkaline phosphatase substrate 5-bromo-4-chloro-3-indolyl phosphate/nitro blue tetrazolium (BCIP/NBT) (Sigma Aldrich) at room temperature for 2 h. The appearance of pinkish brown colonies was monitored over time to avoid oversaturation. The reaction was terminated by removing the substrate solution followed by PBS wash. The bright field images were then acquired using an EVOS FL Cell Imaging System (Thermo Fisher Scientific). To segment spheroids in a bright field image, local contrast was enhanced, followed by a variance and Gaussian filtering and then thresholding (Fig. S1B). In order to obtain the alkaline phosphatase activity/intensity, color deconvolution was done using the vectors (r = 0.65, g = 0.4, b = 0.64). A segmented spheroid with mean alkaline phosphatase activity > 0.5 was considered to be positive (Fig. S1F). These steps were carried out in Fiji^33^.

### Cell proliferation assay

The percentage of cells (cells grown on micropatterns) in the S phase was evaluated by using an in situ cell proliferation kit (Click-iTTM EdU Alexa Fluor 555TM Imaging Kit, Thermofisher scientific) that quantified the incorporation of 5-ethynyl-2ʹ-deoxyuridine (EdU) into cellular DNA. As per the manufacturer's instructions cells were allowed to incorporate 10 μM EdU for 16 h. After EdU incorporation, cells were fixed with 4% paraformaldehyde and permeabilized with 0.5% Triton for 15 min. Following this, cells were incubated with 0.5 mL of Click-iT® reaction cocktail for 30–35 min at room temperature (20–25 °C) and then washed with 1× PBS. Cell nuclei were counterstained with DAPI.

### Image acquisition and analysis

Confocal images were acquired using Nikon A1R laser scanning confocal microscope (Nikon Instruments Inc, Japan), using either × 20 magnification (Plan Apo 20× ELWD, NA 0.8) or 40× magnification (1.25 NA water objective) with identical acquisition settings. Confocal images of 512 × 512 pixels were obtained with an XY optical resolution of 0.3 μm with pinhole size 1 airy unit. In Z dimension each spheroid was scanned up to a depth of 40 μm with a step size of 1 μm. Bright field images were acquired using EVOS FL Cell Imaging System (Thermo Fisher Scientific). Image processing and feature extraction was carried out using custom programs in Fiji^33^.

We used the cytoskeletal protein channel to segment spheroids in a 3D confocal image. We performed a 3D gaussian blurring to smoothen the image and then thresholded to segment spheroids in 3D. We then measure 3D features such as Volume, Sphericity, Moments 1 5, Minimum Feret, Feret, Major Radius, Elongation and Flatness of a bounding ellipsoid, Mean, Min, Max, Standard deviation of Distance from center to surface and used a 2D z-projected image to measure Area, Circularity and Aspect Ratio. In addition, individual nuclei within each colony were segmented. To separate touching nuclei, a 3D euclidean distance transform was performed, thresholded using Otsu method and a 3D watershed algorithm was used to remove touching nuclei.

After segmentation, their nuclear morphology and chromatin organization features were computed as described in a recent study^34^. While a more detailed description of these features can be found in our code repository (https://github.com/SaradhaVenkatachalapathy/colony-image-analysis.git), the features highlighted in this manuscript are described below.

Sphericity of an object is computed as a function of its volume and surface area as described below. Hence a completely spherical colony/nucleus will have a sphericity of value of 1.

$$ Sphericity = \left( {\left( {4\Pi \times Volume^{2} } \right)/\left( {9 \times Surface\;Area^{3} } \right)} \right)^{1/3} . $$

Elongation of an object is the ratio between the semimajor axis and the major axis of the bounding ellipsoid. Therefore elongation of a spherical colony will be one. Flatness of an object is the ratio between the minor axis and the major axis of the bounding ellipsoid. Its value is low when the height of the object is much smaller compared to the projected area of the object. Feret is the maximum dimension (caliper distance) of the object. For a rectangle, the ferret distance will be the length of the diagonal. The minimum radius is the shortest distance between the surface and centroid of the object.

One of the features we used to measure chromatin compaction was the volume of heterochromatin (HC). The heterochromatin was identified using an intensity threshold. The threshold for identifying HC was the sum of mean and 1.5 times Standard deviation of nuclear intensity as described below.

$$ {\text{Threshold}}_{HC} = Mean + 1.5 \times Standard\;Deviation. $$

In addition we also used Harlick’s texture features to describe chromatin organization. For instance the Inverse Difference Moment was used as a measure of local homogeneity.

### Statistical analysis

All statistical analyses and plotting were carried out in R. Hierarchical clustering of day 10 colonies was performed after filtering the highly correlated features. Principal Component Analysis (PCA) was performed on the scaled feature set. The first three principal components were then used to obtain discriminants using Linear Discriminant Analysis (library MASS). Multiple linear regression models (least squares estimation) were used for modeling the relationship of protein expression as a function of the linear discriminant values and time. Diffusion map is a non-linear dimension-reduction method that can also order cells along a continuum. We built a diffusion map to construct the pseudo-trajectory of laterally confined growth and detected branches by clustering^35,36^. We used the first 10 principal components of colony properties across time. The width of the gaussian kernel used was 0.8 and euclidean distance was used as a distance metric. Unless stated otherwise, statistical significance was determined by two-tailed unpaired Student’s t-test. When more than one comparison was done, ANOVA was first performed and if there were significant differences, then Tukey’s HSD test was used to obtain p-values for pairwise comparisons. All experiments were performed at least three times.

In all the boxplots depicted in this manuscript, the box represents the 25th and 75th percentile while the solid line within the box represents the median. The whiskers depict the range excluding the outliers which were identified as those lying outside 1.5 times the interquartile range.

## Supplementary Information

Supplementary Figures.

## Data Availability

The datasets used in the study will be available from the corresponding author on reasonable request.

## Code availability

Code containing the image processing scripts are available https://github.com/SaradhaVenkatachalapathy/colony-image-analysis.git.
