# Supplementary material for: Actomyosin contractility as a mechanical checkpoint for cell state transitions
Source: Sci Rep. 2022 Sep 26;12:16063. doi: 10.1038/s41598-022-20089-8 (PMC9512847; doi:10.1038/s41598-022-20089-8)
Supplement: Supplementary file 1 — Supplementary Figures. [file 41598_2022_20089_MOESM1_ESM.pdf]

Supplementary Information

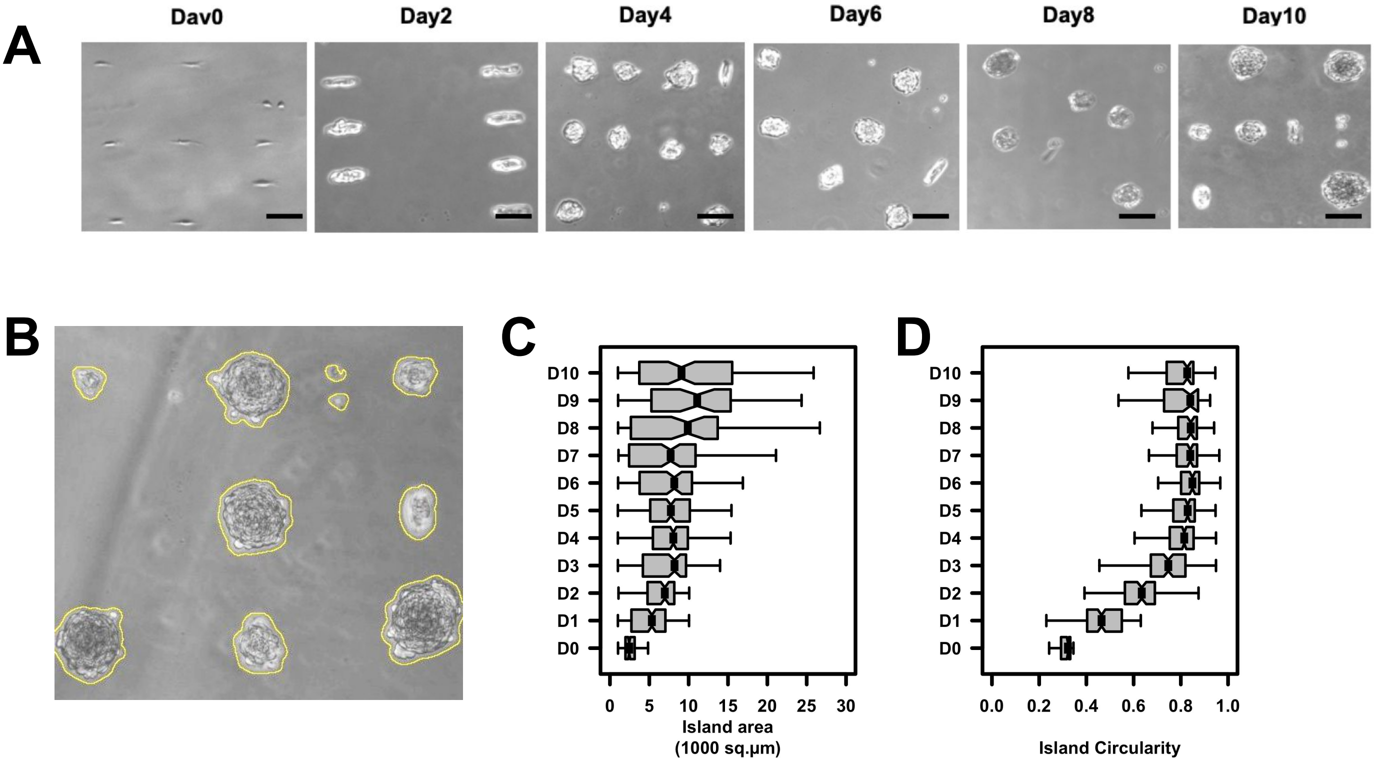

Figure S1. Laterally confined growth of fibroblasts induces cell state transitions: (A) Representative images of HMF3A cells grown on rectangular patterns of laterally confined growth. Scale bar is 100 microns. (B) Identifying spheroids in a brightfield image: yellow lines denote the edge of a single colony. Area (C) and circularity (D) of each island during laterally confined growth. N ~ 100 colonies from 2 biological replicates.

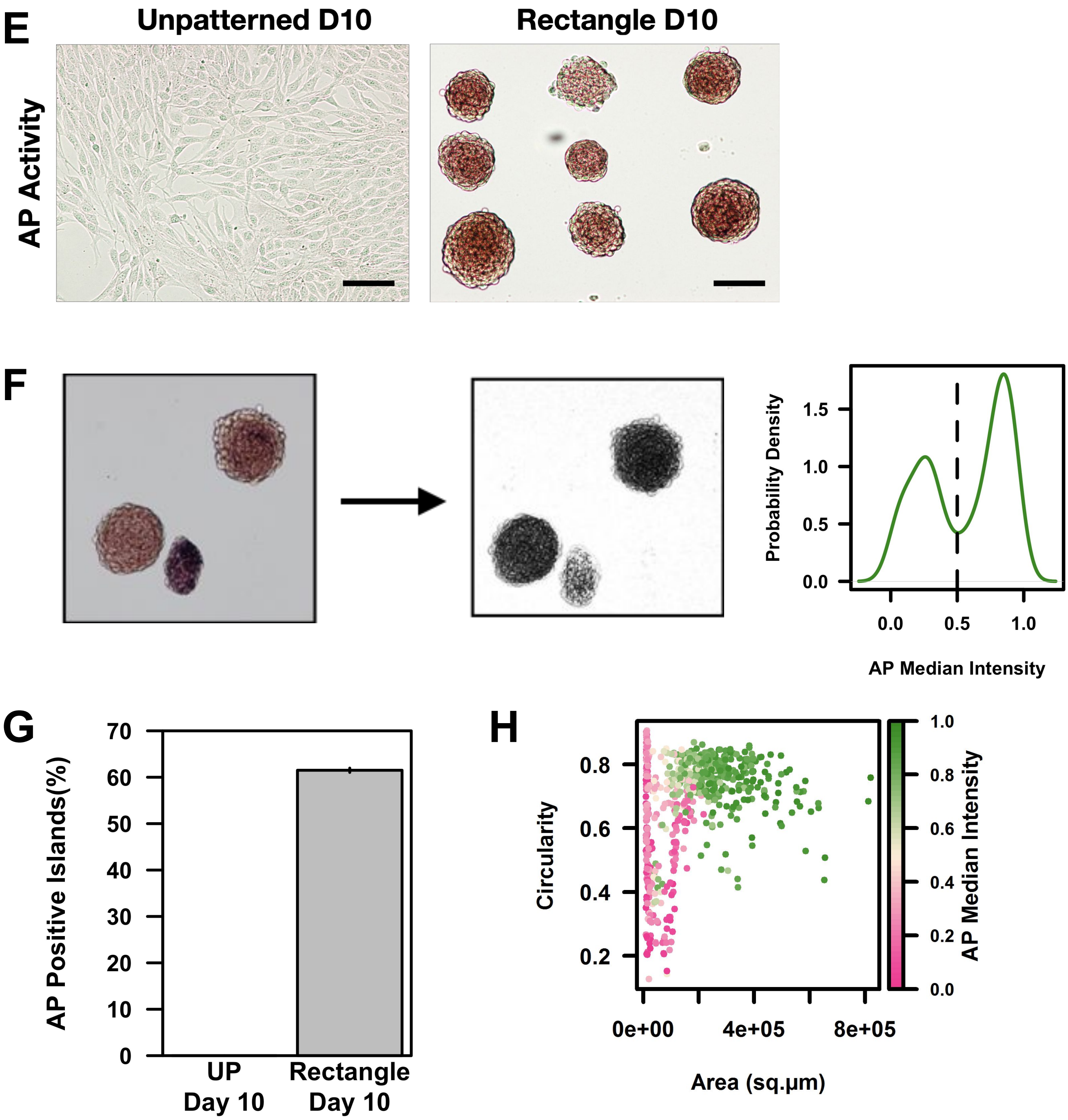

Figure S1. Laterally confined growth of fibroblasts induces cell state transitions: (E) Representative brightfield images showing alkaline phosphatase activity in colonies of HMF3A cells at day 10. Pink/Red is indicative of positive alkaline phosphatase activity. Scale bar is 100 microns. (F) Color deconvolution to obtain AP signal and the threshold for identifying AP positive cells. (G) Fraction of colonies positive for alkaline phosphatase (AP)activity at day 10 (D10). Bar plots represent population means and the segments represent standard error of means. (H) Scatterplot between colony circularity and area after 10 days of culture. Each dot represents one colony, and it is color coded based on the median alkaline phosphatase intensity within the colony. The color key is on the right. N = 1131 colonies from 2 biological replicates.

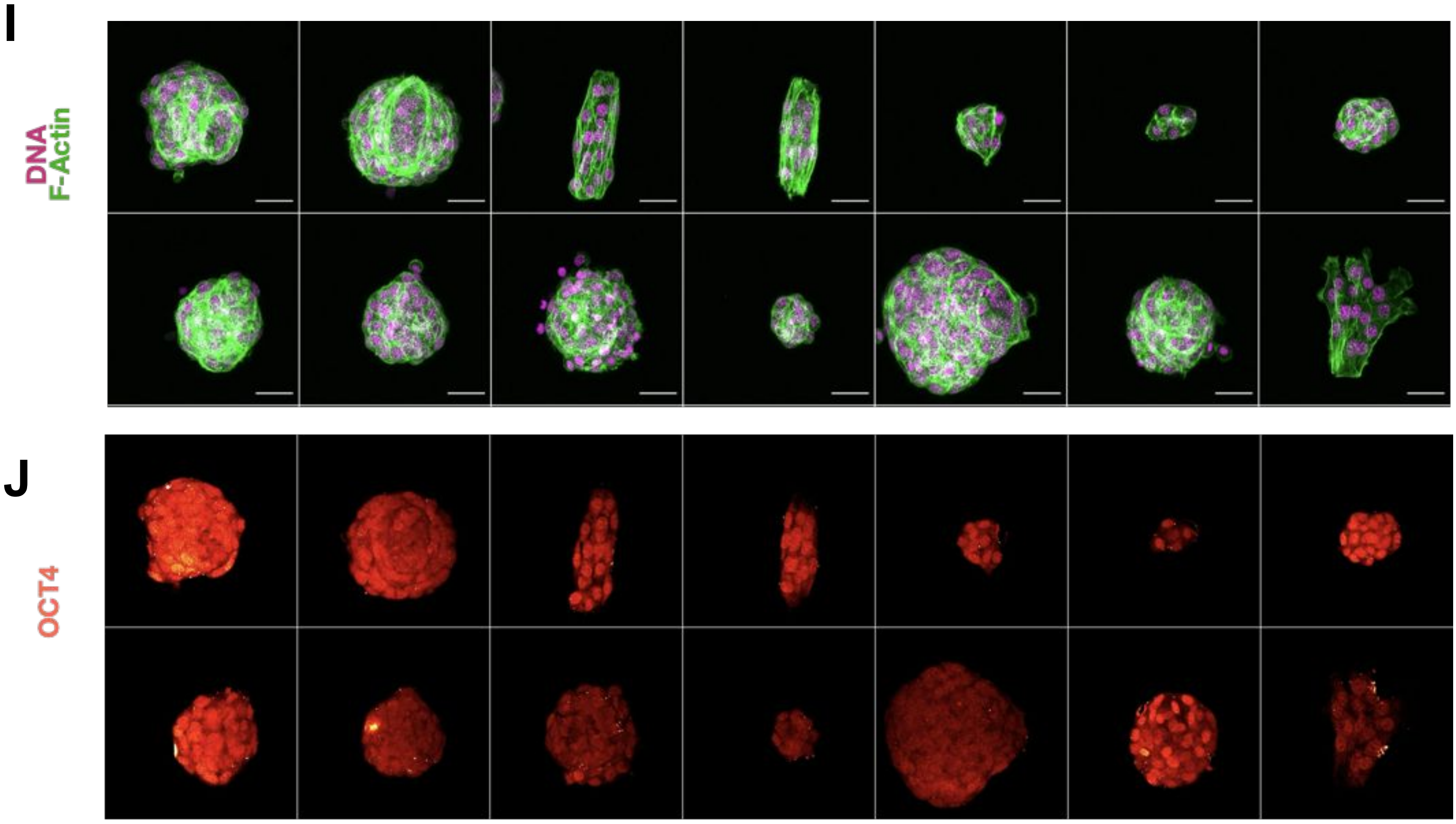

Figure S1. Laterally confined growth of fibroblasts induces cell state transitions: Montage of colonies at day 10 stained for DNA(magenta), F-Actin (green) (I) and Oct4 (heatcolors) (J).

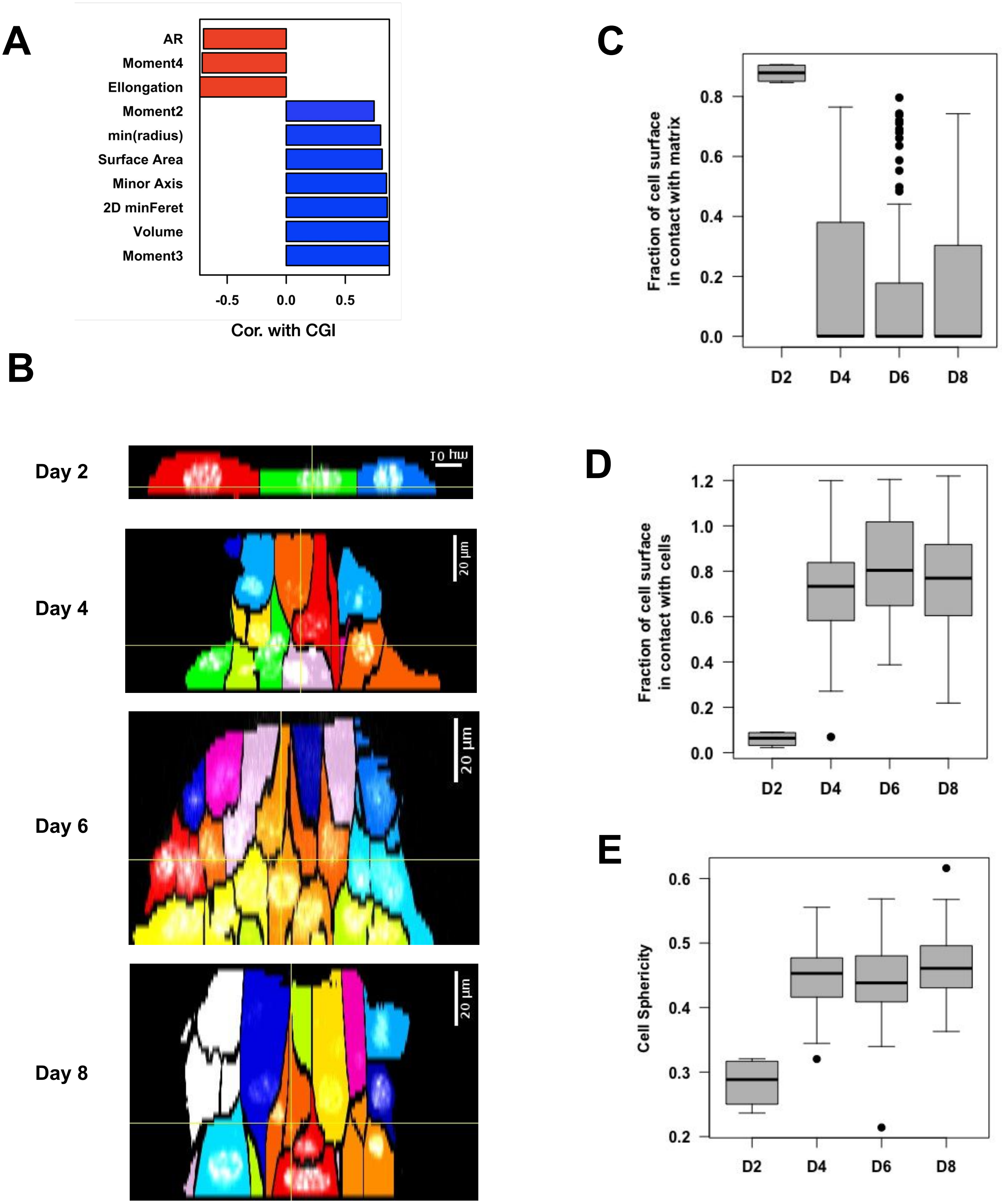

Figure S2 Time course kinetics of reprogramming events demonstrates the coupling between physical and biochemical features: (A) Barplot depicting the top ten colony morphology features with highest spearman rank correlation coefficient (Cor.) with Colony Growth Index. (B) Representative images of XZ slice of colonies during laterally confined growth.DNA (Gray). Each color denotes the 3D voronoi cell of unique nuclei in the colony. Boxplot depicting the fraction of cell surface are that is in contact with the matrix (C), the fraction of the cell's surface area that is in contact with another cell (D) and cell's sphericity (E). These were obtained for one representative colony from 5 biological replicates

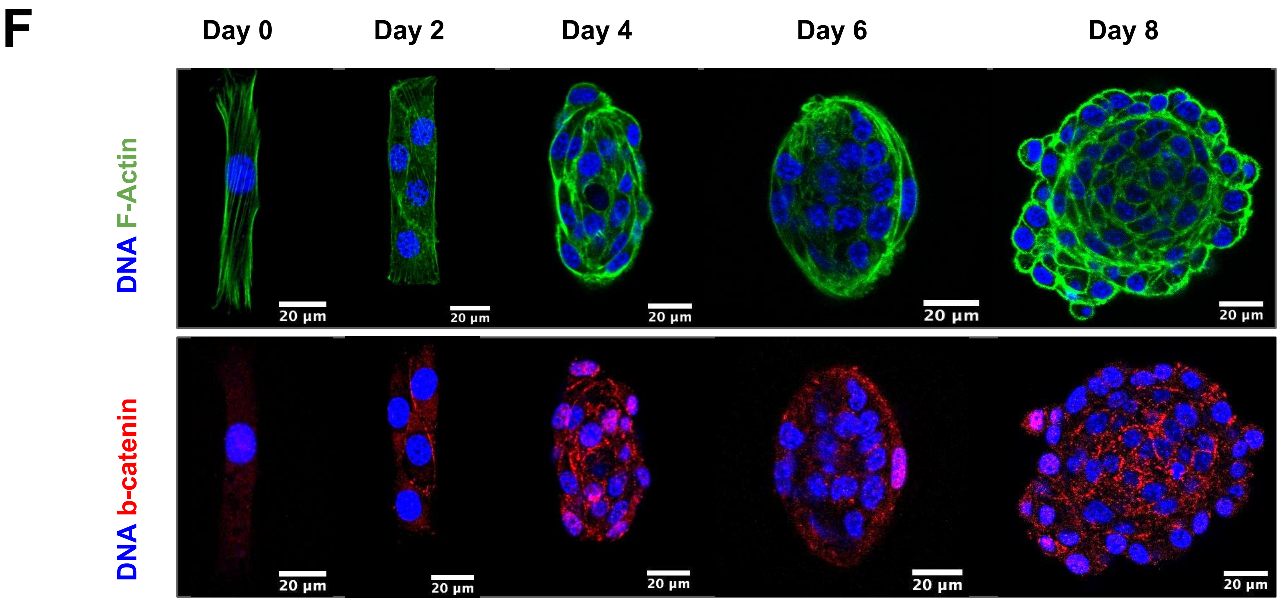

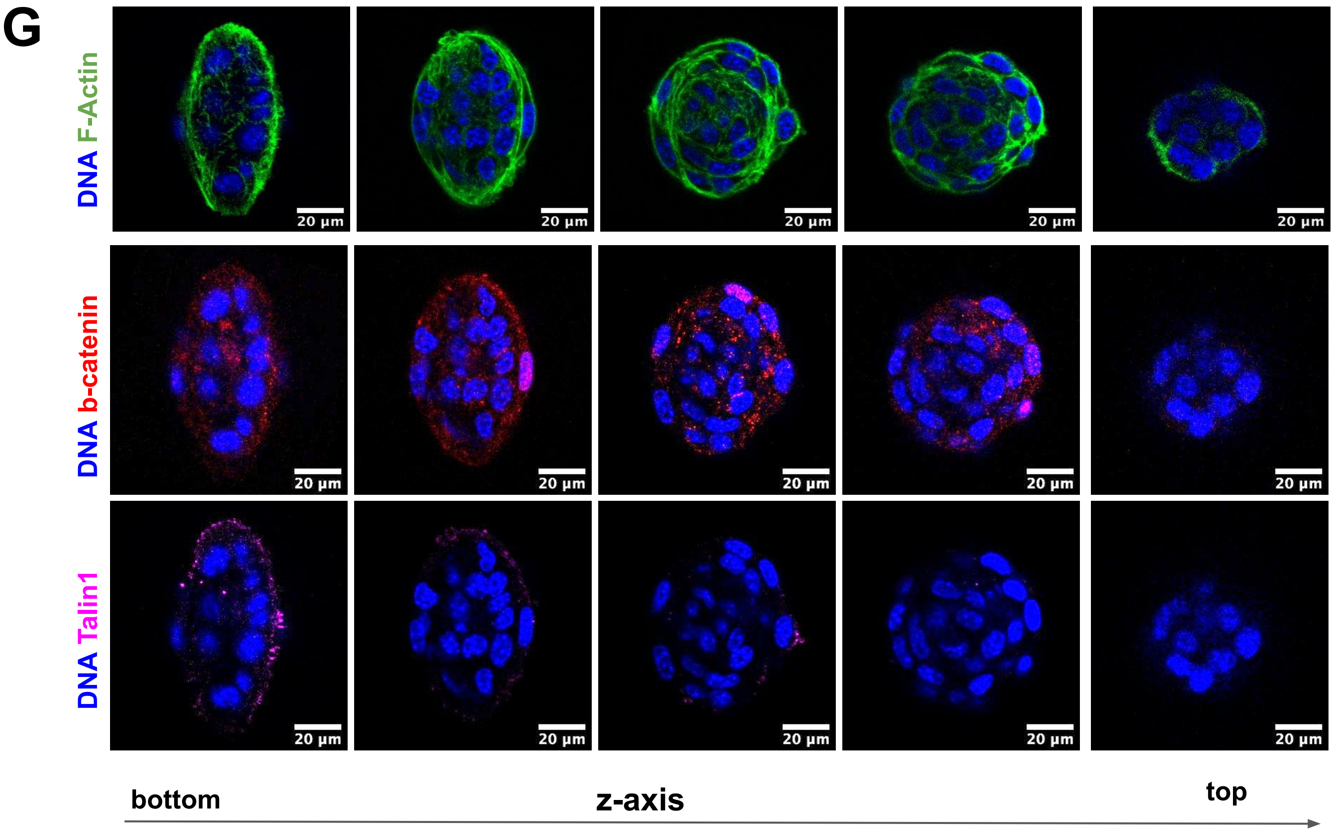

Figure S2 Time course kinetics of reprogramming events demonstrates the coupling between physical and biochemical features: (F) Representative image of colonies during laterally confined growth stained for F-Actin(green) DNA(blue) and B-catenin(red). (G) Representative image of an isotropic colony stained for F-Actin(green) DNA(blue) B-catenin(red) and Talin-1(magenta).

H

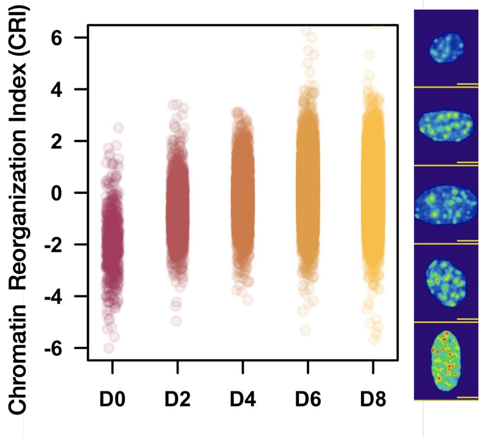

I

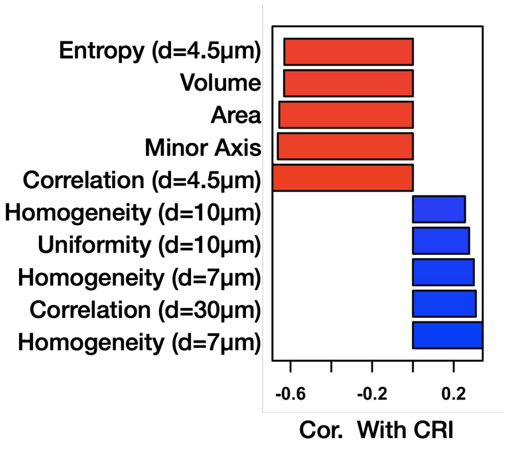

J

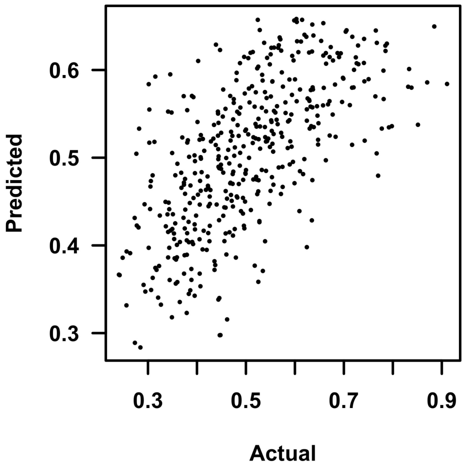

Figure S2 Time course kinetics of reprogramming events demonstrates the coupling between physical and biochemical features: (H) Jitter plot of Chromatin Reorganization Index (CRI) from Day 0 to Day 8. Each dot represents one nucleus. Right: Collage of nuclei labelled for DNA arranged in increasing order of CRI. n=5 biological replicates. (I) Barplot depicting the top ten nuclear features with highest spearman rank correlation coefficient (Cor.) with Chromatin Reorganization Index (CRI). (J) Scatterplot depicting the predicted Oct4 levels by linear regression model versus Actual Oct4 levels. n=3 biological replicates.

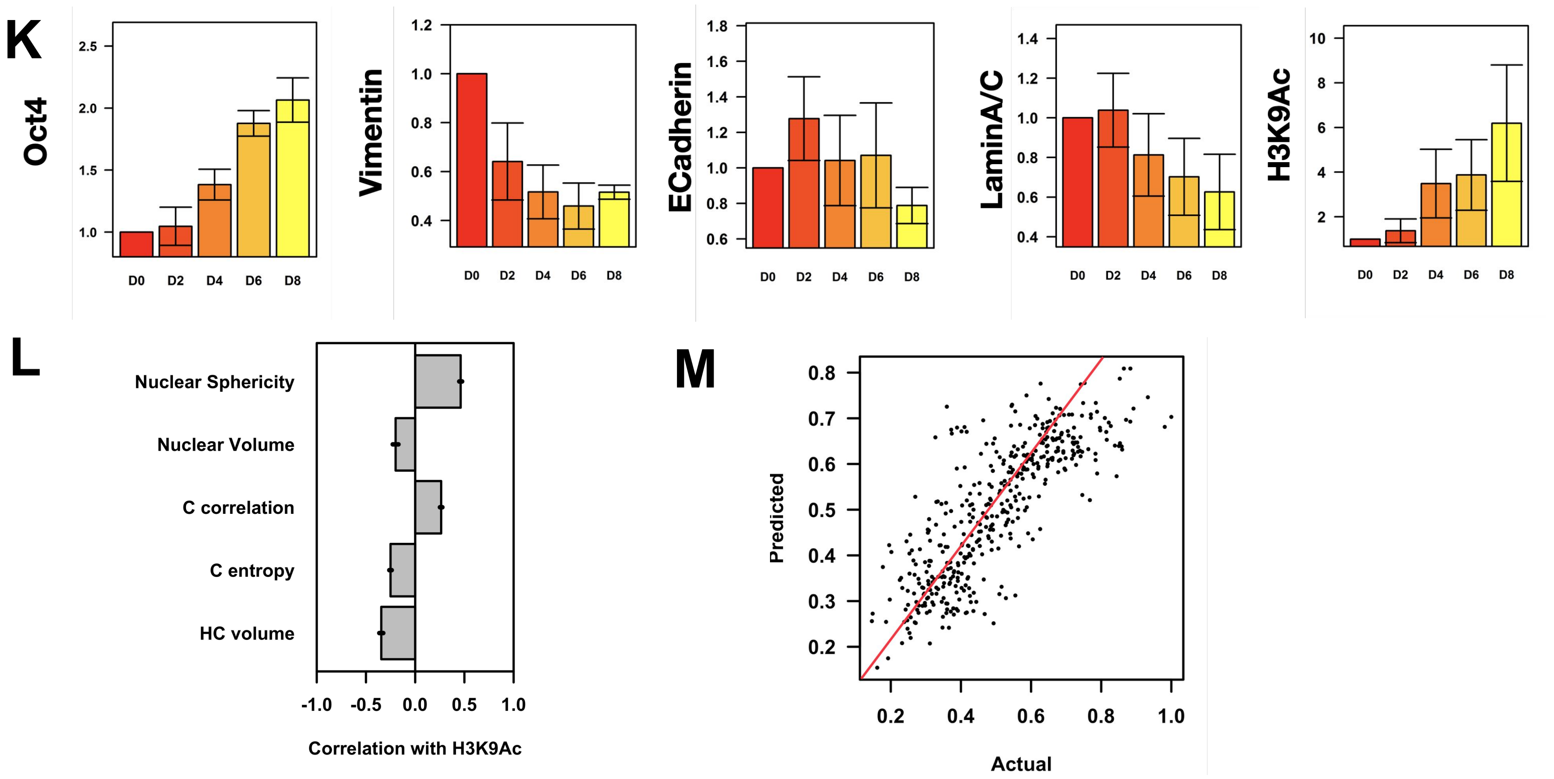

N

	Oct4	Vimentin	E-cadherin	H3K9Ac	Lamin A/C	CRI
R (training)	0.77	0.73	0.80	0.73	0.69	0.86
R.Squared (training)	0.60	0.53	0.65	0.54	0.40	0.74
R (test)	0.72	0.68	0.82	0.68	0.67	0.69
R.Squared (test)	0.51	0.47	0.67	0.46	0.40	0.48

Figure S2 Time course kinetics of reprogramming events demonstrates the coupling between physical and biochemical features: (K) Bar plots depicting the average fold change in the protein expression across time with respect to day 0. The error bars represent the standard error of means across 3 biological replicates. The values are normalized within each biological replicate. One way ANOVA indicated that there were significant changes to protein expression with time ($p<0.01$) (L) Spearman correlation between the cellular H3K9Ac levels and representative nuclear morphology and chromatin organization features. The error bars represent the standard error of means across 3 biological replicates.(M) Scatterplot depicting the predicted Oct4 levels by linear regression model versus Actual Oct4 levels per island. The red line denotes the y=x line. (N) Performance of the linear models. Note the R.Squared value was adjusted for the number of predictors in the model.

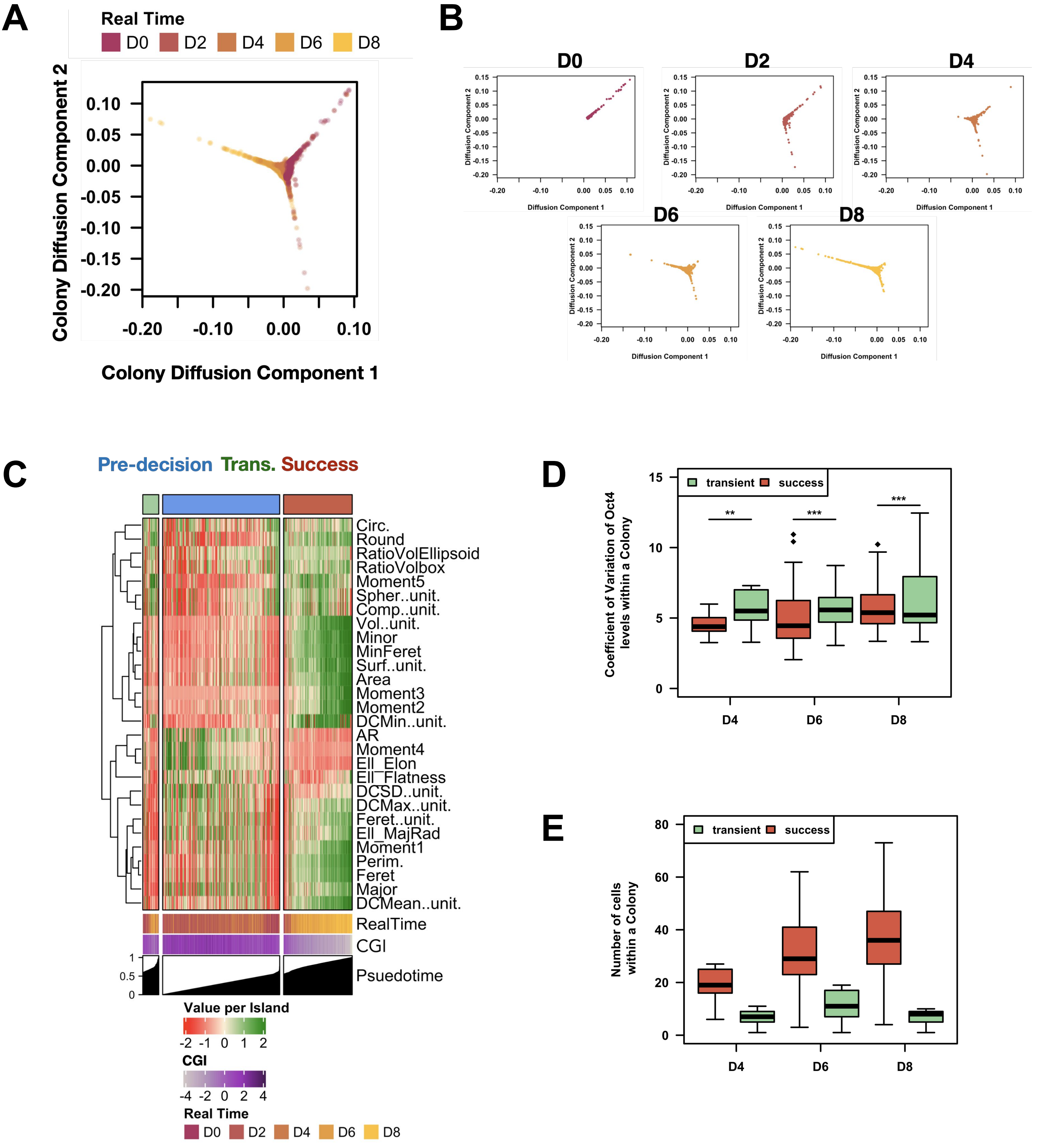

Figure S3 Diffusion analysis reveals stages of laterally confined growth: (A) Diffusion Map of colonies from day 0 to 8 color coded based on the real snapshot time. The color code is along the top margin. (B) Diffusion Maps showing the location of colonies for each time point from days 0 through 8. (C) Heatmap depicting the branch wise changes to the morphological properties of colonies ordered with increasing pseudo time. Note that each column represents one colony. The first column side color bar represents the real time of the colonies between day 0 and 8. The second column color bar represents the colony Growth Index (CGI) values of the colonies. The histogram at the bottom panel depicts the corresponding pseudo time value of the colonies. (D) Boxplot depicting the coefficient of variation of Oct4 levels per nucleus within each colony in colonies belonging to either transient or success branches at Days 4, 6 and 8. (E) Boxplot depicting the number of cells within each colony in colonies belonging to either transient or success branches at Days 4, 6 and 8. Data from 3 biological replicates.

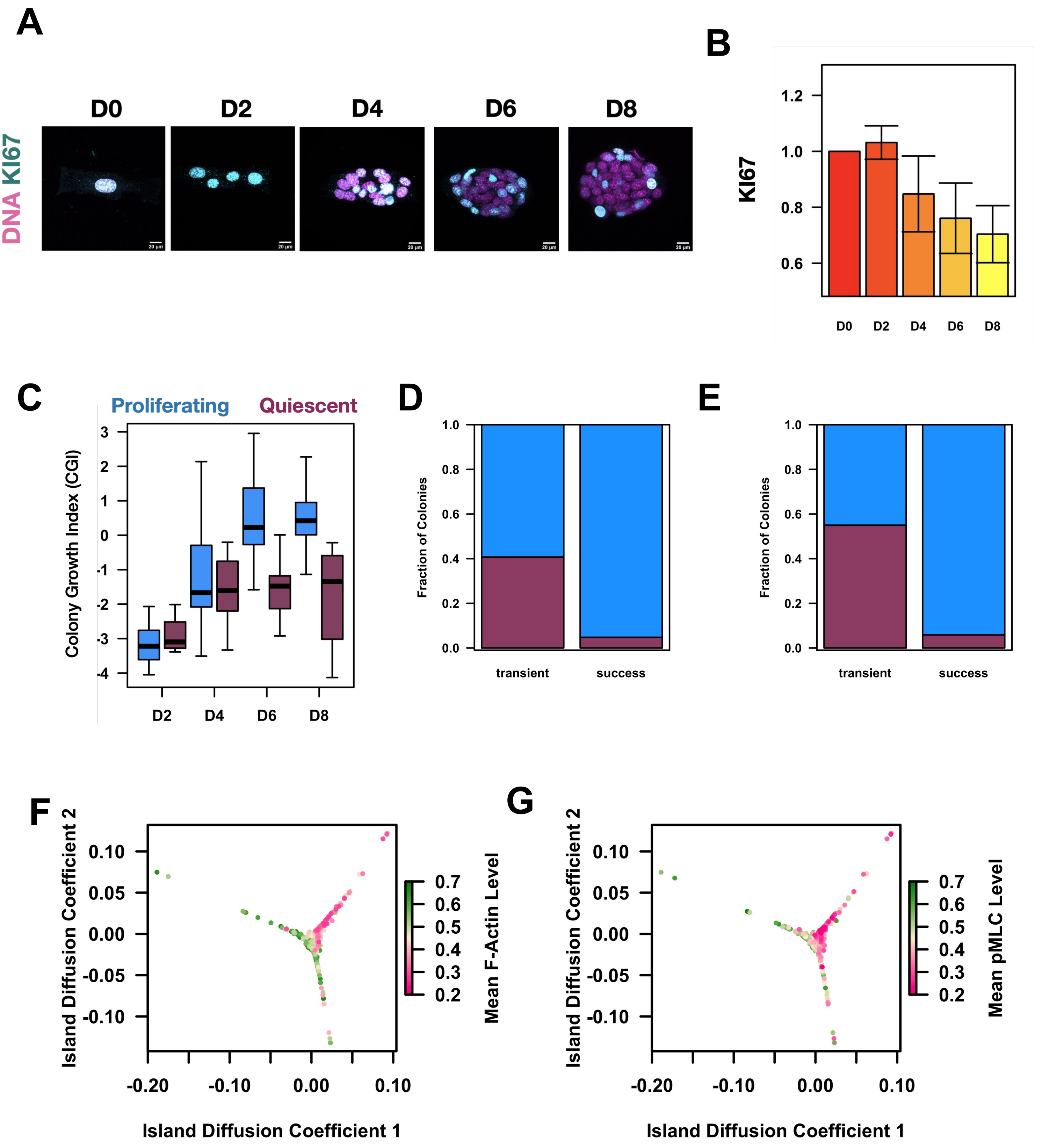

Figure S4 Acto-myosin contractility regulates the cellular decisions during laterally confined growth: (A) Representative images of colonies stained for DNA (Magenta) and Ki67 (Cyan). (B) Bar plot depicting the average fold change in the Ki67 expression across time with respect to day 0. The error bars represent the standard error of means across 3 biological replicates. One way ANOVA indicated that there were significant changes to protein expression with time ($p<0.01$). (C) Boxplot of colony Growth Index of Proliferating(blue) and Quiescent(orchid) across time. n=3 biological replicates. Stacked barplot of the proliferation status of colonies belonging to the success and transient branches at Days 6 (D) and 8 (E) Colonies with at least one Edu positive nucleus are identified to be Proliferating (blue) and the rest are labeled Quiescent (orchid). Data from 3 biological replicates. Diffusion Map of islands color coded with the average F-Actin (F) and pMLC (G) levels per colony. n=3 biological replicates. The values are normalized within each biological replicate.

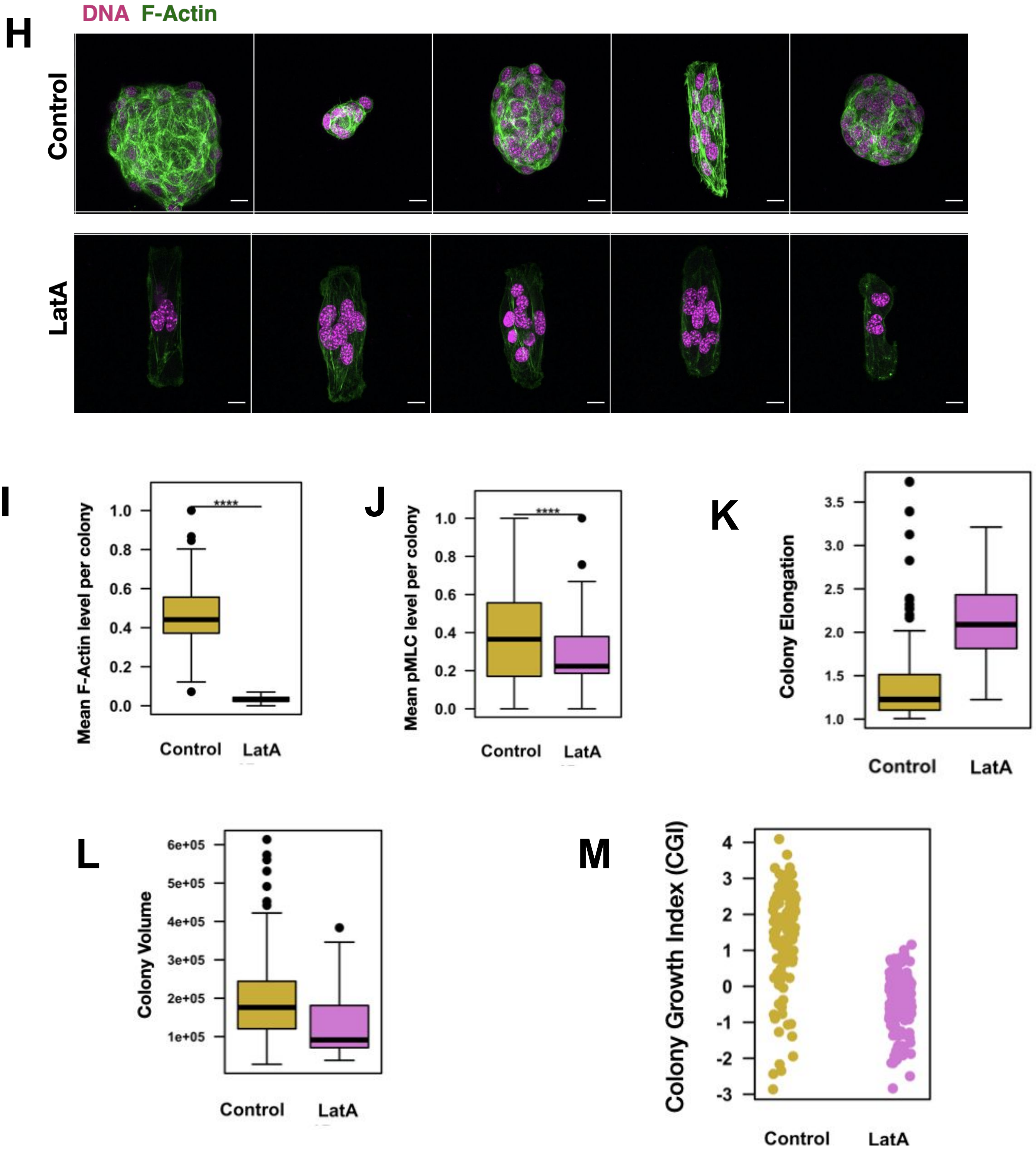

Figure S4 Acto-myosin contractility regulates the cellular decisions during laterally confined growth: (H) Montages of z-projected colonies stained for DNA (magenta) and F-Actin (green) of colonies at day 10 under control culture conditions (yellow, top) and Y Compound (YC) treatment from day 2 to 10 (purple, bottom). Scale bar is 20 microns. Boxplots depicting the Mean F-Actin (I) an pMLC (J) levels per spheroid and Elongation (K) and Volume (L) of colonies under the two culture conditions. The means are significantly different ($p<0.05$). n=150 colonies from 3 biological replicates. (M) Jitter plot depicting the e distribution of colony Growth Index for colonies at day 10 under control culture conditions (yellow) and latrunculin A (LatA) treatment from day 2 to 10 (purple). The means are significantly different ($p<0.05$). Each dot represents one colony. n=150 colonies from 3 biological replicates.

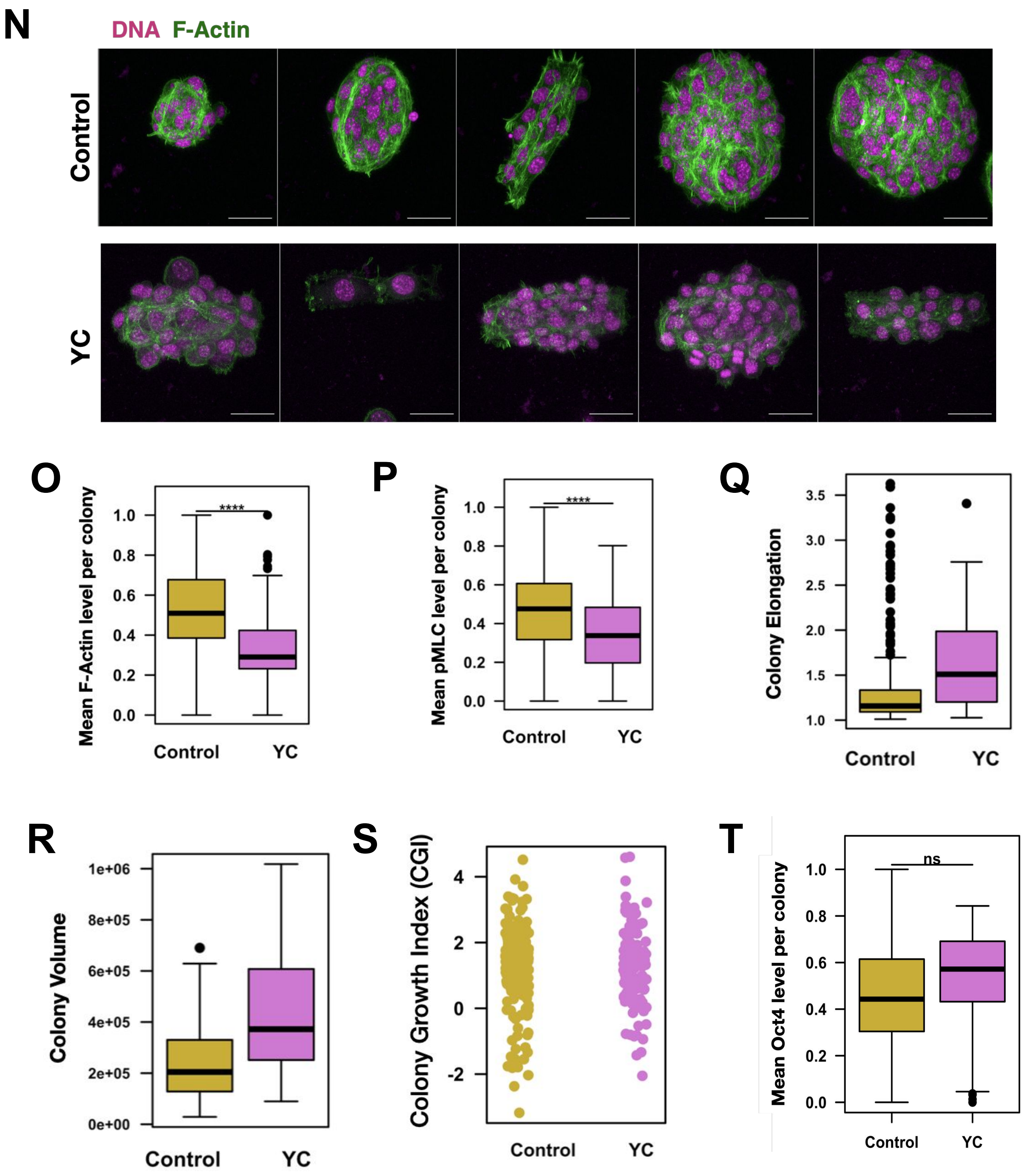

Figure S4 Acto-myosin contractility regulates the cellular decisions during laterally confined growth: (N) Montages of z-projected colonies stained for DNA (magenta) and F-Actin (green)of colonies at day 10 under control culture conditions (yellow, top) and Y Compound(YC) treatment from day 2 to 10 (purple, bottom). Scale bar is 20 microns. Boxplots depicting the Mean F-Actin (O) and pMLC (P) levels per spheroid and Elongation (Q) and Volume (R) of colonies under the two culture conditions. The means are significantly different ($p<0.05$). n=150 colonies from 3 biological replicates. (S) Jitter plot depicting the distribution of Colony Growth Index for colonies at day 10 under control culture conditions (yellow) and Y Compound (YC) treatment from day 2 (purple). The means are significantly different ($p<0.05$). Each dot represents a colony. n=150 colonies from 3 biological replicates. (T) Mean Oct4 Levels of control and Y-Compound (YC) treated colonies at day 10.

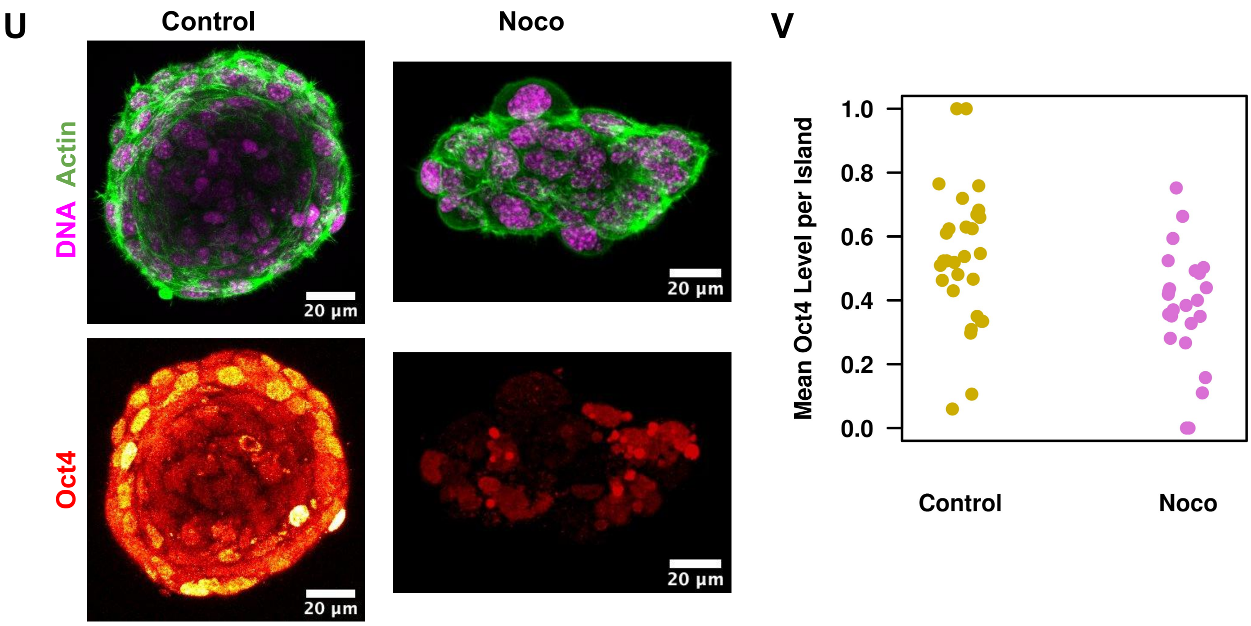

Figure S4 Acto-myosin contractility regulates the cellular decisions during laterally confined growth: (U) Representative images of control and nocodazole (Noco) treated colonies at day 10. F-Actin (Green), DNA (Magenta)and Oct4 (Heatcolors). Scale bar = 20µm (V) Mean Oct4 Levels of control and nocodazole (Noco) treated colonies that belong to the success branch at day 10. $p<0.05$ n= 30 colonies from 3 biological replicates.
